# Interactions among nutrients govern the global grassland biomass–precipitation relationship

**DOI:** 10.1073/pnas.2410748122

**Published:** 2025-04-11

**Authors:** Philip A. Fay, Laureano A. Gherardi, Laura Yahdjian, Peter B. Adler, Jonathan D. Bakker, Siddharth Bharath, Elizabeth T. Borer, W. Stanley Harpole, Erika Hersch-Green, Travis E. Huxman, Andrew S. MacDougall, Anita C. Risch, Eric W. Seabloom, Sumanta Bagchi, Isabel C. Barrio, Lori Biederman, Yvonne M. Buckley, Miguel N. Bugalho, Maria C. Caldeira, Jane A. Catford, QingQing Chen, Elsa E. Cleland, Scott L. Collins, Pedro Daleo, Christopher R. Dickman, Ian Donohue, Mary E. DuPre, Nico Eisenhauer, Anu Eskelinen, Nicole Hagenah, Yann Hautier, Robert W. Heckman, Ingibjörg S. Jónsdóttir, Johannes M. H. Knops, Ramesh Laungani, Jason P. Martina, Rebecca L. McCulley, John W. Morgan, Harry Olde Venterink, Pablo L. Peri, Sally A. Power, Xavier Raynaud, Zhengwei Ren, Christiane Roscher, Melinda D. Smith, Marie Spohn, Carly J. Stevens, Michelle J. Tedder, Risto Virtanen, Glenda M. Wardle, George R. Wheeler

**Affiliations:** ^a^United States Department of Agriculture, Agricultural Research Service, Grassland, Soil, and Water Lab, Temple, TX 76502; ^b^Department of Environmental Sciences, Policy, and Management, University of California, Berkeley, CA 94720; ^c^Instituto de Investigaciones Fisiológicas y Ecológicas Vinculadas a la Agricultura-Consejo Nacional de Investigaciones Científicas y Técnicas, Cátedra de Ecología, Facultad de Agronomía, Universidad de Buenos Aires, Ciudad Autónoma de Buenos Aires C1417DSE, Argentina; ^d^Department of Wildland Resources and the Ecology Center, Utah State University, Logan, UT 84322; ^e^School of Environmental and Forest Sciences, University of Washington, Seattle, WA 98195; ^f^Department of Ecology, Evolution, and Behavior, University of Minnesota, St. Paul, MN 55108; ^g^German Centre for Integrative Biodiversity Research, Halle-Jena-Leipzig, Leipzig 04103, Germany; ^h^Department of Physiological Diversity, Helmholtz Center for Environmental Research, Leipzig 04318, Germany; ^i^Martin Luther University Halle-Wittenberg, Halle (Saale) 06108, Germany; ^j^Department of Biological Sciences, Michigan Technological University, Houghton, MI 49930; ^k^Department of Ecology and Evolutionary Biology, University of California, Irvine, CA 92697; ^l^Department of Integrative Biology, University of Guelph, Guelph, ON N1G 2W1, Canada; ^m^Swiss Federal Institute for Forest, Snow and Landscape Research WSL, Birmensdorf 8903, Switzerland; ^n^Centre for Ecological Sciences, Indian Institute of Science, Bangalore 560012, India; ^o^Faculty of Environmental and Forest Sciences, Agricultural University of Iceland, Reykjavík 112, Iceland; ^p^Department of Ecology, Evolution, and Organismal Biology, Iowa State University, Ames, IA 50011; ^q^Co-Centre for Climate + Biodiversity + Water, School of Natural Sciences, Trinity College Dublin, Dublin 2, Ireland; ^r^Center for Applied Ecology “Prof. Baeta Neves”-Research Network in Biodiversity and Evolutionary Biology, School of Agriculture, University of Lisbon, Lisbon 1349-017, Portugal; ^s^Forest Research Centre, Associate Laboratory TERRA, School of Agriculture, University of Lisbon, Lisbon 1349-017, Portugal; ^t^Department of Geography, King’s College London, London WC2B 2BG, United Kingdom; ^u^Fenner School of Environment & Society, Australian National University, Canberra, ACT 2600, Australia; ^v^Senckenberg Museum for Natural History Görlitz, Görlitz D-02826, Germany; ^w^Ecology, Behavior & Evolution Department, University of California San Diego, La Jolla, CA 92103; ^x^Department of Biology, University of New Mexico, Albuquerque, NM 87131; ^y^Instituto de Investigaciones Marinas y Costeras, Facultad de Ciencias Exactas y Naturales, Universidad Nacional de Mar del Plata-Consejo Nacional de Investigaciones Científicas y Técnicas, Mar del Plata B7600WAG, Argentina; ^z^Desert Ecology Research Group, School of Life and Environmental Sciences, The University of Sydney, NSW 2006, Australia; ^aa^Zoology, School of Natural Sciences, Trinity College Dublin, Dublin 2, Ireland; ^bb^MPG Ranch, Missoula, MT 59833; ^cc^Institute of Biology, Leipzig University, Leipzig 04103, Germany; ^dd^Ecology and Genetics Unit, University of Oulu, Oulu FI-90014, Finland; ^ee^Department of Zoology and Entomology, Mammal Research Institute, University of Pretoria, Pretoria 400364, South Africa; ^ff^Department of Biology, Ecology and Biodiversity Group, Utrecht University, Utrecht 3584 CH, The Netherlands; ^gg^Department of Biology, University of North Carolina, Chapel Hill, NC 27599; ^hh^Department of Integrative Biology, University of Texas at Austin, Austin, TX 78712; ^ii^Life and Environmental Sciences, University of Iceland, Reykjavik 102, Iceland; ^jj^Department of Health and Environmental Sciences, Xi’an Jiaotong-Liverpool University, Suzhou 215123, China; ^kk^Department of Environmental Science and Policy, Marist College, Poughkeepsie, NY 12601; ^ll^Department of Biology, Texas State University, San Marcos, TX 78666; ^mm^Department of Plant and Soil Sciences, University of Kentucky, Lexington, KY 40546; ^nn^Department of Environment and Genetics, La Trobe University, Bundoora, VIC 3083, Australia; ^oo^Department of Biology-Wildness, Biodiversity, and Ecosystems Under Change, Vrije Universiteit Brussel, Brussels 1050, Belgium; ^pp^Universidad Nacional de la Patagonia Austral-Instituto Nacional de Tecnología Agropecuaria-Consejo Nacional de Investigaciones Científicas y Técnicas, Rio Gallegos CP 9400, Santa Cruz, Argentina; ^qq^Hawkesbury Institute for the Environment, Western Sydney University, Penrith, NSW 2751, Australia; ^rr^Sorbonne Université, Université de Paris- Cité, Université Paris-Est Créteil, Institut de Recherche pour le Développement, Centre National de la Recherche Scientifique, Institut National de Recherche pour l'Agriculture, l'Alimentation et l'Environnement, Institut d'Ecologie et des Sciences de l'Envrionnement de Paris, Paris 75005, France; ^ss^College of Ecology, Lanzhou University, Lanzhou City 730000, China; ^tt^Gansu Gannan Grassland Ecosystem National Observation and Research Station, Maqu County 747300, Gansu Province, China; ^uu^Department of Biology, Colorado State University, Fort Collins, CO 80523; ^vv^Department of Soil and Environment, Swedish University of Agricultural Sciences, Uppsala 75007, Sweden; ^ww^Lancaster Environment Centre, Lancaster University, Lancaster LA1 4YQ, United Kingdom; ^xx^Centre for Functional Biodiversity, School of Life Sciences, University of KwaZulu-Natal, Pietermaritzburg 3209, South Africa; ^yy^School of Life and Environmental Sciences, ARC Training Centre in Data Analytics for Resources and Environments, The University of Sydney, Sydney, NSW 2006, Australia; ^zz^School of Biological Sciences, University of Nebraska-Lincoln, Lincoln, NE 68588

**Keywords:** primary productivity, precipitation, diversity, grasslands

## Abstract

Ecosystems are experiencing changing global patterns of mean annual precipitation (MAP) and enrichment with multiple nutrients that potentially colimit plant biomass production. In grasslands, mean aboveground plant biomass is closely related to MAP, but how this relationship changes after enrichment with multiple nutrients remains unclear. We hypothesized the global biomass–MAP relationship becomes steeper with an increasing number of added nutrients, with increases in steepness corresponding to the form of interaction among added nutrients and with increased mediation by changes in plant community diversity. We measured aboveground plant biomass production and species diversity in 71 grasslands on six continents representing the global span of grassland MAP, diversity, management, and soils. We fertilized all sites with nitrogen, phosphorus, and potassium with micronutrients in all combinations to identify which nutrients limited biomass at each site. As hypothesized, fertilizing with one, two, or three nutrients progressively steepened the global biomass–MAP relationship. The magnitude of the increase in steepness corresponded to whether sites were not limited by nitrogen or phosphorus, were limited by either one, or were colimited by both in additive, or synergistic forms. Unexpectedly, we found only weak evidence for mediation of biomass–MAP relationships by plant community diversity because relationships of species richness, evenness, and beta diversity to MAP and to biomass were weak or opposing. Site-level properties including baseline biomass production, soils, and management explained little variation in biomass–MAP relationships. These findings reveal multiple nutrient colimitation as a defining feature of the global grassland biomass–MAP relationship.

## Significance

Understanding how multiple interacting nutrients regulate the global relationship between mean annual precipitation and aboveground biomass is crucial for forecasting how ecosystem functioning will be altered by ongoing global changes. We fertilized with nitrogen, phosphorus, and potassium plus micronutrients in all combinations in 71 grasslands representing a global precipitation gradient. The grassland biomass–precipitation relationship became steeper with an increasing number of added nutrients. Increases in steepness corresponded to the form of interaction among added nitrogen and phosphorus. We found weak evidence that variation in plant species diversity mediated changes in the biomass–precipitation relationship. Multiple nutrient colimitation, particularly by nitrogen and phosphorus, is a defining feature of grassland biomass–precipitation relationships, and crucial to predicting grassland responses to global change.

**Fig. 1.**
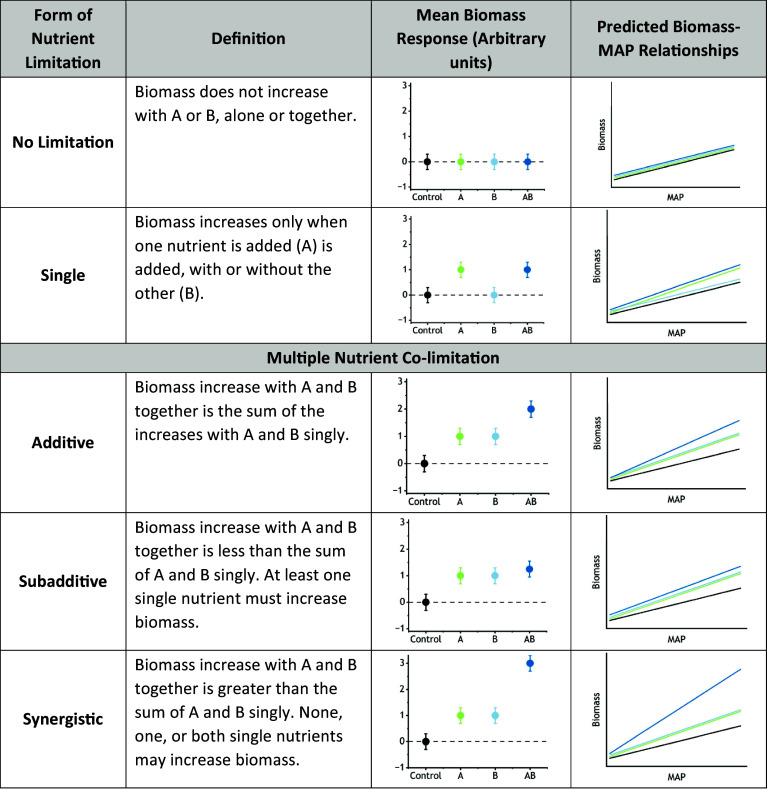
Conceptual framework for predicting changes in the steepness of the global mean aboveground biomass–MAP relationship in response to fertilization with hypothetically limiting nutrients A, B, and A together with B (AB). A and B represent any two nutrients that potentially limit biomass production. The mean response of biomass across sites to fertilization defines the number of nutrients limiting aboveground biomass and the form of interactions among colimiting nutrients—additive, subadditive, or synergistic. The increase in slope of the grassland biomass–MAP relationship is predicted from the mean biomass response. Limitation forms are generalized from ref. 10. Application of the rubric is detailed in *SI Appendix, Extended Methods*.

Terrestrial ecosystems across the globe are experiencing changes in mean annual precipitation (MAP), with MAP increasing in some regions and decreasing in others (1). Concurrently, many ecosystems are increasingly enriched with multiple elemental nutrients (2) including nitrogen (N), phosphorus (P), and potassium (K), which frequently colimit plant aboveground biomass production (3–13), a major component of ecosystem primary productivity (14). In grasslands, site-level mean aboveground biomass increases with increasing ecosystem MAP—the biomass–MAP relationship (15–23). At a global scale, the grassland biomass–MAP relationship emerges from across considerable variation in other factors regulating biomass production including, topography, soils, grazing, and other management (24). Theory predicts greater nutrient limitation of biomass production with increasing MAP (17, 19), reflecting higher demand for nutrients required to maintain plant carbon metabolism and water balance (13, 25, 26). Thus, fertilizing with limiting nutrients should result in a biomass–MAP relationship with a steeper slope (Fig. 1) (27–29). However, to what extent the increase in steepness depends on the number or identity of added nutrients or interactions among them is poorly understood. Clarifying the role of multiple nutrient interactions is necessary to forecast how interacting global change drivers—climate change and nutrient enrichment—will affect global patterns in energy flow, primary productivity, and ecosystem services. These processes are critical to societal efforts to mitigate and adapt to the impacts of global change drivers (2, 30, 31).

We propose a new framework—the “Multiple Nutrient Colimitation” Hypothesis—predicting how the number of limiting nutrients and interactions among colimiting nutrients influence the global grassland biomass–MAP relationship. This hypothesis holds that the effects of fertilization on the steepness of the biomass–MAP relationship: 1) increase with the number of added nutrients and attendant reduction in multiple nutrient limitation and 2) corresponds to the form of interaction among multiple nutrients (10). This hypothesis extends previous concepts of the controls on the grassland biomass–MAP relationship (17, 19, 27–29) by explicitly accounting for the number of limiting nutrients and how colimiting nutrients interact (Fig. 1). This idea also builds on previous findings of widespread multiple nutrient colimitation of productivity in grasslands (12, 32) and other ecosystems (10, 33).

Interactions among colimiting nutrients occur in several forms defined by the responses in aboveground biomass when a site is fertilized with nutrients individually and in combination (Fig. 1) (10, 34). Synergistic colimitation is present when the response of biomass to fertilizing with multiple nutrients is greater than the sum of the single nutrient responses and is common in terrestrial ecosystems (10, 34). Additive colimitation occurs when the response to multiple nutrients equals the sum of the single nutrient responses. Subadditive colimitation occurs when the response is less than the sum of the single nutrient responses; this form is uncommon (10, 34) and may reflect intensification of another limiting factor. Single nutrient limitation or no responses to the added nutrients are also possible. The Multiple Nutrient Colimitation hypothesis predicts that fertilizing grasslands with multiple nutrients should cause a synergistic increase in slope of the biomass–MAP relationship across sites where mean biomass production is synergistically colimited, and corresponding responses in slope across sites where colimitation is additive, subadditive, or where limitation is by a single nutrient. However, the correspondence between the increase in slope of the global grassland biomass–MAP relationship and the form of nutrient limitation has not been evaluated, primarily because the multiple nutrient enrichment experiments needed to directly test these effects in grasslands spanning a globally relevant range of MAP have only recently become available (35).

Plant community diversity plays a central role in mediating biomass production (36–42). Thus, variation in plant community diversity is expected to mediate the relationships of MAP and added nutrients to biomass production. For example, with increasing MAP, sites should increase in species richness (43–47) and favor faster-growing, more productive species (48) which may also have higher nutrient requirements (28, 49). At the same time, higher-MAP communities may also be more susceptible to the synergistic effects of adding multiple limiting nutrients, including amplified plant species losses (29, 50, 51), stronger dominance (52–55), or other deviations from the regional species pool (56, 57). Therefore, we predict greater mediation of MAP and nutrient effects on biomass by plant community diversity in synergistically colimited grasslands than in grasslands with other forms of nutrient limitation. Resolving whether the form of nutrient limitation alters mediation of the global grassland biomass–MAP relationship by plant diversity is crucial for forecasting how climate change and eutrophication impact the provision of biomass-related ecosystem services in grasslands.

Here, we test the Multiple Nutrient Colimitation hypothesis by analyzing the relationship of site mean aboveground biomass production (hereafter, “biomass”) to site MAP across 71 grasslands (*SI Appendix*, Table S1) in a global multiple nutrient fertilization experiment, the Nutrient Network (58). These grasslands were distributed across six continents (*SI Appendix*, Fig. S1) and spanned 12 to 991 g m^−2^ aboveground biomass, 167 to 1,823 mm y^−1^ MAP, −3.3 to 24.1 °C mean annual temperature, and 0 to 4,241 m elevation (*SI Appendix*, Table S1). The sites included native and planted grasslands with varying soil textures and soil nutrient contents. Of the 71 sites, 26 were reported by site investigators to be under active management, mostly burning or grazing (17 sites). The form of management was unspecified for the remainder. Management contributes to variation in relationships among precipitation, biomass production, and plant diversity (59). Thus, we evaluated the effects of multiple nutrient fertilization on the biomass–MAP relationship across broadly representative global precipitation gradients and realistic sources of complexity in grassland ecosystem structure and function. Standardized multiple nutrient fertilization treatments were conducted for 4 to 14 y (*SI Appendix*, Table S1 and *Abbreviated Methods*). All sites fertilized 5 m × 5 m plots once each year with 10 g m^−2^ each of N, P, and K in factorial combination in a randomized blocks design with at least three replicates. This fertilization rate exceeds global mean deposition rates for these nutrients (0.4 to 1.5 g m^−2^ y^−1^) (60–62). The K treatment included micronutrients in years 1 and 10 (Kµ). Peak live aboveground biomass and plant species cover were determined annually. From the cover, we derived effective species richness (eH), evenness, and beta diversity (βplot). Site MAP was the average of the precipitation accrued between the annual biomass harvests (*SI Appendix, Climate Variables*). Biomass and the variables derived from cover in each plot were averaged across years by plot to correspond in temporal scale to site MAP (*SI Appendix, Extended Methods*).

Our analysis addressed three primary research questions about grassland biomass—MAP—nutrient interactions: 1) Does the global biomass–MAP relationship become steeper with increased number of added nutrients? 2) Does the increased steepness of the biomass–MAP relationship correspond to the form of nutrient limitation? 3) Does the form of nutrient limitation alter the mediation of MAP and nutrient effects on biomass by community diversity?

## Results

### Does the Global Biomass–MAP Relationship Become Steeper with Increased Number of Added Nutrients?

**Fig. 2.**
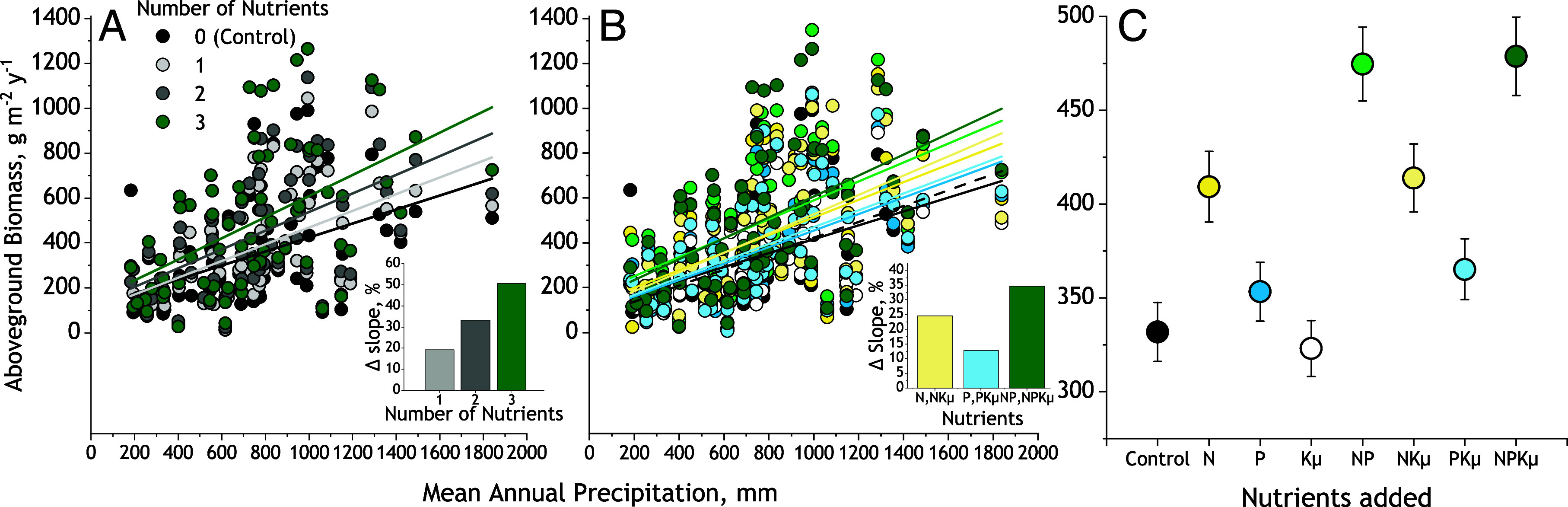
Responses of the global mean biomass—MAP relationship to fertilization with single N, P, and potassium with micronutrients (Kµ). (*A*) The biomass–MAP relationships for treatments fertilizing with 0, 1, 2, or 3 nutrients. Inset: the percent increase in linear regression slope relative to unfertilized controls. (*B*) the biomass–MAP relationships for treatments fertilizing with N, P, and Kµ in factorial combinations. Nutrient treatments are color-coded as in Panel *C*. (*Inset*) The percent increase in linear regression slopes relative to unfertilized controls for N, P, and NP treatments averaged across levels of Kµ. (*C*) Mean ± SE of aboveground biomass across all 71 sites for the factorial N, P, and Kµ fertilization treatments. See Table 1 for linear mixed model analyses and *SI Appendix*, Table S2 for linear regression equations.

Across all 71 sites, the biomass of unfertilized control plots significantly increased with MAP (slope = 0.31, R^2^ = 0.19, *P* < 0.0001, *SI Appendix*, Table S2). As hypothesized, fertilization increased the steepness of the biomass–MAP relationship, and this increase was more pronounced when greater numbers of nutrients were added (Fig. 2*A*; MAP × Number of Nutrients *P* < 0.0001, Table 1). Fertilization with all three nutrients–N, P, and Kµ together—increased the slope of the biomass–MAP relationship by 51% compared to the baseline slope for control plots (Fig. 2 *A*, *Inset* and *SI Appendix*, Table S2). The increase in slope was smaller, 33%, for fertilization with pairs of nutrients (NP, NKµ, or PKµ), and smallest, 19%, for fertilization with single nutrients (N, P, or Kµ alone; Fig. 2 *A*, *Inset*).

**Table 1.** Linear mixed model F statistics, degrees of freedom (dfs), and *P*-values testing the effects of the number of nutrients added, N, P, potassium with micronutrients (Kµ), MAP, and their interactions on aboveground biomass across all 71 sites

Mixed model effects	F(dfs)	*P*-value
Number (#) of Nutrients		
# of Nutrients	72.5(3,125)	**<0.0001**
MAP	27.9(1, 69)	**<0.0001**
MAP × # of nutrients	8.4(3,131)	**<0.0001**
Factorial nutrient combinations		
N	260.7(1,447)	**<0.0001**
P	63.6(1,447)	**<0.0001**
N*P	7.0(1,447)	**0.0084**
K	0.3(1,447)	0.5604
N*K	0.1(1,447)	0.7981
P*K	0.6(1,447)	0.4586
N*P*K	0.8(1,447)	0.3680
MAP	28.3(1, 69)	**<0.0001**
MAP*N	19.2(1,460)	**<0.0001**
MAP*P	4.3(1,460)	**0.0376**
MAP*N*P	0.0(1,460)	0.8554
MAP*K	3.6(1,460)	0.0577
MAP*N*K	0.2(1,460)	0.6657
MAP*P*K	0.2(1,460)	0.6919
MAP*N*P*K	0.2(1,460)	0.6541

Model effects and statistics for number of nutrients added correspond to Fig. 2*A*, and for factorial nutrient combinations correspond to Fig. 2 *B* and *C*. *P*-values are in bold font when <0.05.

Fertilizing with greater numbers of nutrients increased the steepness of the biomass–MAP relationship across all sites primarily because slopes increased under fertilization with N (*P* < 0.0001) and P (<0.0001, Fig. 2*B* and Table 1). Fertilizing with N alone increased the slope by 26% compared to unfertilized plots (Fig. 2 *B*, *Inset* and *SI Appendix*, Table S2), while fertilizing with P alone increased the slope by only 19%. In contrast, fertilizing with N and P together increased the slope 36%. N and P did not interact with MAP to influence biomass (*P* = 0.86, Table 1). This signifies that fertilizing with N and P together caused an additive increase in the steepness of the biomass–MAP relationship (Fig. 1). Fertilizing with Kµ together with N and P caused slight increases in the slope of the biomass–MAP relationship (Fig. 2*B* and *SI Appendix*, Table S2) but there was little indication that Kµ interacted with MAP, N, or P (0.06 < *P* < 0.86, Table 1). These findings support the hypothesis that increasing numbers of nutrients, and particularly fertilizing with N and P, increases the steepness of the grassland biomass–MAP relationship.

### Does the Increased Steepness of the Biomass–MAP Relationship Correspond to the Form of Nutrient Limitation?

Unexpectedly, across all 71 sites the additive increase in steepness of the global biomass–MAP relationship did not correspond to the effects of N and P fertilization on the mean biomass production across all sites. Adding N and P together synergistically increased mean aboveground biomass (Fig. 2*C*; N × P *P* = 0.008, Table 1). Biomass rose 43% compared to 23% for adding N alone and 6% for P alone. The synergistic mean biomass response corresponded instead to a synergistic increase in the intercept of the global biomass–MAP relationship rather than in the slope (*SI Appendix*, Table S2). However, the absence of Kµ effects on global mean biomass (0.37 < *P* < 0.80, Table 1) was consistent with the absence of interaction of Kµ with MAP.

**Fig. 3.**
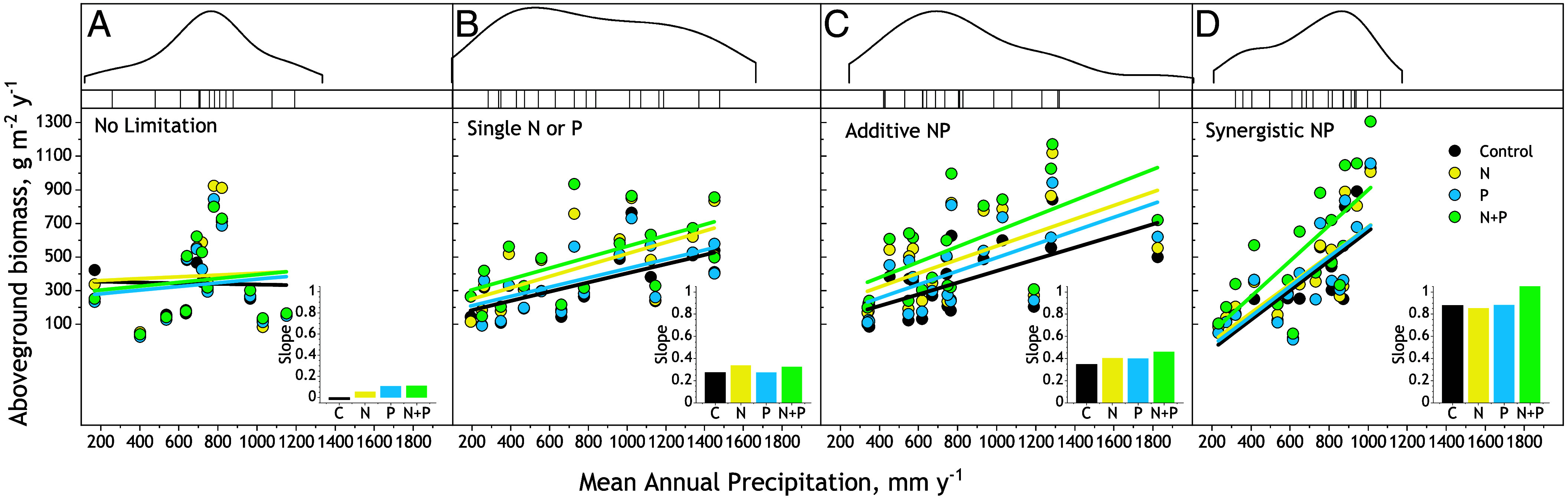
Aboveground biomass in relation to MAP for sites classified by form of response to N and P fertilization. (*A*) No Limitation, (*B*) Limited by Single N or P, (*C*) Additive limitation by N and P, (*D*) Synergistic limitation by N and P). Response forms are defined in Fig. 1. N and P treatments are averaged across levels of Kµ fertilization. *Insets* depict the slopes for unfertilized control (C), N, P, and N together with P. *Upper* panels are kernel-smoothed MAP distributions for the sites each form of limitation. See Table 2 for linear mixed model analyses and *SI Appendix*, Table S2 for linear regression equations.

Instead, increases in steepness of the biomass–MAP relationship corresponded with the effects of N and P fertilization across sites expressing the same form of limitation by N and P (*SI Appendix*, Table S2). For example, across the 15 sites classified as synergistically colimited by N and P (Fig. 3*D*), adding N and P together caused a synergistic increase in slope (20%, MAP × N × P *P* = 0.04, Table 2 and *SI Appendix*, Table S2), while fertilization with N or P individually caused little effect on slope (Fig. 3 *D*, *Inset*). The correspondence between increases in slope and form of nutrient limitation continued in sites classified as additively colimited by N and P (21 sites, Fig. 3*C*), single-nutrient limited (13 sites by N, 3 by P, Fig. 3*B*). The correspondence of limitation form with slope response even extended to sites not limited by N or P (15 sites, Fig. 3*A*), where slopes did not respond to adding N or P (Table 2 and *SI Appendix*, Table S2).

**Table 2.** Linear mixed model F statistics and *P*-values testing the effects of N, P, and MAP, and their interactions on aboveground biomass for sites classified by form of limitation by N and P (Fig. 3)

	No limitation		Single		Additive		Synergistic	
Mixed model effects	F(dfs)	*P*-value	F(dfs)	*P*-value	F(dfs)	*P*-value	F(dfs)	*P*-value
N	4.9(1,281)	**0.0278**	109.6(1, 89)	**<0.0001**	204.3(1,133)	**<0.0001**	55.0(1,96)	**<0.0001**
P	0.5(1,281)	0.4598	8.8(1, 89)	**0.0039**	55.4(1,133)	**<0.0001**	48.3(1,96)	**<0.0001**
N*P	0.0(1,281)	0.9862	0.5(1, 89)	0.4739	0.5(1,133)	0.4837	30.4(1,96)	**<0.0001**
MAP	0.3(1, 13)	0.6091	14.3(1, 14)	**0.0020**	8.7(1, 19)	**0.0081**	13.3(1, 13)	**0.0030**
MAP*N	2.0(1,281)	0.1627	4.6(1,99)	**0.0344**	8.8(1,143)	**0.0035**	1.7(1,93)	0.1917
MAP*P	2.3(1,281)	0.1297	0.0(1,99)	0.8339	4.4(1,143)	**0.0383**	5.0(1,93)	**0.0279**
MAP*N*P	0.6(1,281)	0.4454	0.2(1,99)	0.6687	0.0(1,143)	0.9207	4.4(1,93)	**0.0397**

*P*-values are in bold font when <0.05. N and P effects and interactions are across levels of Kµ.

**Fig. 4.**
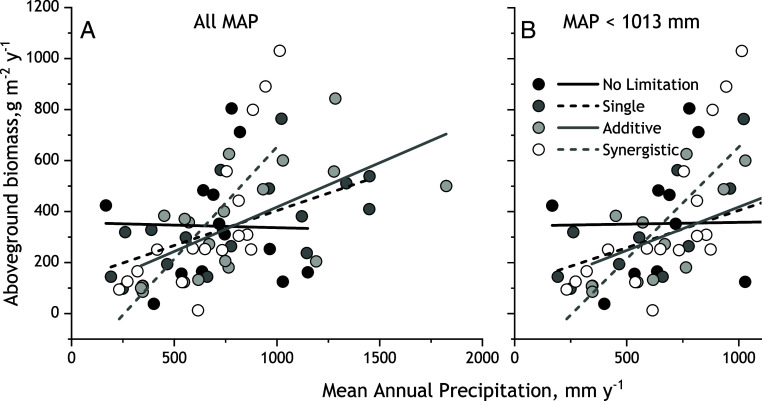
Baseline global biomass–MAP relationships defined by unfertilized controls across sites classified by form of response to N and P fertilization (Fig. 3). (*A*) Biomass–MAP relationships across the MAP range spanned by all 71 sites. (*B*) Biomass–MAP relationships for sites with MAP up to 1,013 mm. See Table 3 for linear mixed models analyses and *SI Appendix*, Table S2 for linear regression equations.

The correspondence of increases in slopes of biomass–MAP relationships to the form of nutrient limitation emerged from substantial differences among limitation forms in the strength of the baseline biomass–MAP relationships for unfertilized grassland (MAP × form *P* < 0.0059, Table 3). For example, across synergistically colimited sites, the baseline unfertilized biomass–MAP relationship was steepest (slope = 0.88) and explained the most variation (R^2^ = 0.49) among the four limitation classes (Fig. 4*A*). In contrast, across No Limitation sites, baseline aboveground biomass was uncorrelated with MAP (*SI Appendix*, Table S2). Baseline biomass–MAP relationships across single nutrient-limited and across additive N-P colimited sites fell between these extremes (slopes = 0.27 to 0.46; R^2^ = 0.27 to 0.37).

**Table 3.** Linear mixed model F statistics and *P*-values testing the effects of the form of nutrient limitation (Form), MAP and their interaction on unfertilized aboveground biomass (Fig. 4)

	All MAP		MAP < 1,013 mm	
Mixed model effects	F(dfs)	*P*-value	F(dfs)	*P*-value
Form	0.7(3, 56)	0.5621	0.3(3, 43)	0.8014
MAP	14.7(1, 56)	**0.0003**	12.0(1, 43)	**0.0012**
MAP*Form	4.6(3, 56)	**0.0059**	0.9(3, 43)	0.4260

*P*-values are in bold font when <0.05.

Synergistically colimited sites spanned a lower range of MAP (up to 1,013 mm, Fig. 3*D*) than sites in the other limitation categories. This could explain the steeper baseline biomass–MAP relationship because in drier regions primary production is increasingly controlled by precipitation inputs (63). When we standardized the range of MAP in the limitation categories by excluding sites with >1,013 mm MAP (the maximum of the synergistic sites) from the Additive, Single, and No Limitation categories (Fig. 4*B*), baseline slopes did not differ among limitation forms (MAP × form *P* = 0.43, Table 3 and *SI Appendix*, Table S2). Importantly, the correspondence between the form of limitation and the increases in slope with addition of N and P remained (*SI Appendix*, Fig. S2). Other site-level factors potentially affecting how fertilization with N and P increased the steepness and strength of biomass–MAP relationships, including management, latitude, elevation, mean annual temperature and the texture, and nutrient content of soils did not differ in occurrence or magnitude among limitation categories (*SI Appendix*, Table S3). These findings show that the effect of fertilization with N and P on the steepness of the biomass–MAP relationship corresponded to the form of nutrient limitation across variation in other factors that can potentially influence this relationship.

### Does the Form of Nutrient Limitation Alter Mediation of MAP and Nutrient Effects on Biomass by Community Diversity?

**Fig. 5.**
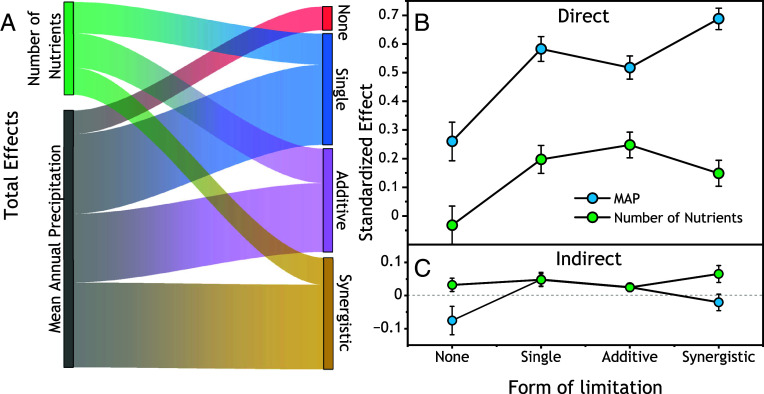
Summary of the standardized effects of nutrient addition and MAP on aboveground biomass production from structural equation models fit (Table 4) across grassland sites in each of four forms of nutrient limitation. (*A*) Sankey plot depicting Total effects (direct + indirect) of number of nutrients and MAP (*Left* side) mapped onto each form of nutrient limitation (*Right* side). The widths of the links depict the magnitude of each total effect for each limitation form. (*B*) Direct and (*C*) indirect effects (±SE) of MAP and nutrient addition for each limitation form. Indirect effects represent community mediation of MAP and nutrient effects on biomass, and combine paths through effective species richness, species evenness, and beta diversity (*SI Appendix*, Fig. S3).

Contrary to our hypothesis, we found little evidence that plant community diversity mediated relationships of MAP to biomass. Structural equation models successfully partitioned indirect (“community mediated”) and direct effects of fertilization and MAP on biomass (*P* > 0.59, Table 4 and *SI Appendix*, Fig. S3). Across sites in each form of nutrient limitation, MAP was always the largest driver of aboveground biomass. Total (direct + indirect) MAP effects ranged from 0.18 across No Limitation sites to 0.67 across synergistic colimitation sites and were 2 to 3 times greater than total nutrient effects (Fig. 5*A* and *SI Appendix*, Table S4). Total MAP effects were almost entirely explained by direct effects (Fig. 5*B*). Indirect, community-mediated effects of MAP were near 0 (0.08 < *P* < 0.41) for No Limitation and Synergistic limitation sites and small (5 to 8% of total effects, *P* < 0.045, Fig. 5*C* and *SI Appendix*, Table S4) for Single and Additive limitation sites. Indirect effects of MAP were weak because the individual paths linking the diversity variables to MAP and biomass were either not significant or offsetting (*SI Appendix*, Fig. S3). In contrast, indirect nutrient effects on biomass ranged from 9 to 30% of total effects (*P* < 0.045, Fig. 5*C* and *SI Appendix*, Table S4) and were largest in Synergistic limitation sites, consistent with large decreases in means of eH, Evenness, and βplot across all sites (*SI Appendix*, Fig. S4).

**Table 4.** Fit statistics for structural equation models fit across grassland sites assigned to four forms of nutrient limitation by nitrogen and phosphorus (Fig. 5)

	Fit statistic		
Kind of limitation	X2 (*P*-value)	RMSEA	PCF
	Thresholds		
	*P* > 0.05	<0.06	>0.05
No Limitation	1.07 (0.5860)	0.0000	0.6962
Single	0.25 (0.8803)	0.0000	0.9284
Additive	0.83 (0.6597)	0.0000	0.8128
Synergistic	0.06 (0.9711)	0.0000	0.9823

χ^2^: Chi-square test. RMSEA:Rootmean square error. PCF:Probability of close fit.

## Discussion

Anthropogenic global changes are causing long-term changes in MAP while concurrently enriching ecosystems with multiple potentially limiting nutrients (1, 2). These changes will have significant consequences for aboveground plant biomass production, a key component of primary productivity, global carbon cycling, and many ecosystem services. Our findings largely supported the Multiple Nutrient Colimitation hypothesis. They demonstrated that the global grassland biomass–MAP relationship became steeper as the number of added nutrients increased (question 1) and that the increased steepness of the biomass–MAP relationship corresponded to the form of nutrient limitation (question 2). However, we found little evidence that the form of nutrient limitation affected mediation of the biomass–MAP relationship by the combined effects of eH, evenness, or βplot (question 3). These findings provide robust evidence that the grassland biomass–MAP relationship is limited by multiple nutrients (17, 19) and reveal the key importance of the number, identity, and interactions among the limiting nutrients.

It is well established that the predominant forms of nutrient limitation of biomass production—single limitation, additive colimitation, synergistic colimitation, or no nutrient limitation—are widespread in the world’s grasslands (9, 10, 12, 32, 51). These findings build on this foundation by revealing the correspondence of the increased steepness of the biomass–MAP relationship to the mean response to fertilizing with single and multiple nutrients, in particular N and P (Figs. 2 and 3). Steeper biomass–MAP relationships in response to fertilization are consistent with greater nutrient uptake to meet greater demand by plant metabolism (13, 25, 26) and with steeper and more variable within-site temporal biomass–precipitation relationships (64). The present findings extend the generality of our previous findings of widespread site-level synergistic colimitation of grassland biomass production by N and P (12, 32) by including nearly 30 more sites with many fertilized for over a decade (vs. 4 to 7 y), allowing more forms of nutrient interactions to be resolved.

Steeper biomass–MAP relationships in response to fertilizing nutrient-limited sites with N and P indicated that increases across sites in mean water availability increasingly translated into greater plant biomass production (16, 19, 22, 23, 65, 66). However, the biomass–MAP relationships displayed considerable scatter in all fertilization treatments. For example, MAP explained only about 20% of the variation in plant biomass across all 71 sites in the control treatment (Fig. 2*B*), and fertilization treatments yielded only modest increases in variation explained by MAP (25 to 30%; *SI Appendix*, Table S2). The relatively high baseline variation seen in our study may be unsurprising for sites spanning multiple continents, large differences in plant species assemblages, and varying management, soils, latitude, and aspects of climate other than MAP (*SI Appendix*, Tables S1 and S3). However, in the synergistically colimited sites, MAP explained about 50% of the variation in biomass (*SI Appendix*, Table S2), approached levels previously reported (65). The synergistically colimited grasslands may be more similar to each other in the mechanisms linking biomass production to water availability than are sites in the other limitation forms we identified.

Our findings ran counter to our prediction that plant community change would mediate the biomass–MAP relationship (36, 67, 68). We found weak evidence that combined changes in effective species richness, evenness, or beta diversity mediated the biomass–MAP relationship, despite stronger community mediation of fertilization effects on biomass, which aligns with previous findings that fertilization reduces compositional stability (57, 69, 70) and synergistic global responses in two out of three measures of species diversity in response to fertilization with N and P (*SI Appendix*, Fig. S4). The weak signal for community diversity mediation of the biomass–MAP relationship is also consistent with findings that large compositional shifts in global grasslands with plant invasion were unable to explain changes in biomass over the last several decades (24). However, including these diversity metrics in our analysis, even if their net effect was small, was still important. SEMs resolved a significant biomass–MAP relationship in sites not limited by N or P (Fig. 5*B* and *SI Appendix*, Fig. S3 and Table S4) where analysis not accounting for diversity (Fig. 3*A*) did not. This finding highlights the importance of accounting for changing plant community attributes when evaluating factors governing the global biomass–MAP relationship.

Several underlying mechanisms may explain the weak community mediation finding. Fertilization effects on species diversity within sites may be poor predictors of responses across larger spatial gradients (71) because the MAP gradient encompasses large, potentially nonlinear diversity changes (47) while within-site responses are limited by the local species pool. In addition, longer time periods may be required to detect plant community mediation than the 4 to 14 y of observation we had available here (36, 67). Biomass and diversity responses increased through 11 y of fertilization with N, P and K (72), so community mediation may emerge when more sites accumulate more years of fertilization (41). Finally, we did not consider abundance-weighted composition metrics (42) or functional diversity, which decreased with nutrient addition across some Nutrient Network sites (73) and in some instances may better predict ecosystem function than species-based metrics alone (74, 75). However, biomass gains following fertilization can be explained by plant species that persist following fertilization rather than by replacement (53–55, 72). Further analysis of plant compositional and functional dynamics in grasslands differing in form and strength of single and multiple nutrient limitation is a promising area for future research.

We continue to find little evidence for global-average limitation of grassland biomass by Kµ, alone or in combination with N and P, although a few individual sites are Kµ limited (12, 32). The infrequency of K-limitation may reflect several factors. K and micronutrients are broadly abundant in many surface soils, and K is strongly retained by plants compared to N or P (13). Plant tissue K concentration is tightly regulated because of its critical role in metabolite transport and water balance. However, K is also susceptible to loss from the rooting zone by leaching, which may be more prevalent in sites expressing K-limitation (13, 62). We also found no evidence for differences among limitation forms in occurrence or magnitude of site-level factors (*SI Appendix*, Table S3) that might influence fertilization effects on biomass–MAP relationships. The prevalence of N and P limitation varies with latitude, temperature, and fire frequency (12, 76, 77), and background soil nutrient levels and texture can influence retention and uptake of water and nutrients (78–81). Resolving the contributions of these factors will be aided by the network’s growing dataset of responses to multiple nutrient fertilization.

Our findings point to a critical need for better understanding of edaphic mechanisms causing single and multiple nutrient limitation in grasslands. Because water is necessary for biogeochemical processes, mechanisms likely center on ways that water availability influences nutrient availability (82, 83). The number of limiting nutrients and the form of interaction between colimiting nutrients may depend on the alignment of water availability and nutrient availability. Water availability interacts with soil parent material, microbial processing, biogeochemical cycling, stoichiometry, and plant uptake of the nutrients (84–88). For example, Vázquez et al. (89) found that synergistic increases in aboveground biomass production resulted in part from enhanced N and P uptake and retention. A comprehensive spatial model incorporating mechanistic drivers of single and multiple nutrient limitation is needed to link with productivity models to predict global scale responses of ecosystem productivity to changing precipitation and eutrophication.

The core findings of this study support our Multiple Nutrient Colimitation hypothesis. The effects of fertilization on the steepness of the global biomass–MAP relationship: 1) increases with the number of added nutrients and attendant reduction in multiple nutrient limitation, and 2) corresponds to the form of interactions among colimiting nutrients, particularly N and P. This critical, globally relevant insight into the regulation of grassland productivity can be exploited to predict the interactive effects of eutrophication and hydrologic intensification on grassland productivity and related ecosystem services. Applying this insight will require predicting the number and form of interaction among multiple limiting nutrients across the world’s grasslands, and developing a more general understanding of magnitude and extent to which plant community change mediates grassland productivity–precipitation relationships and of the edaphic mechanisms controlling nutrient limitation.

## Abbreviated Methods

### Fertilizer Treatments.

Each site applied fertilizers following the Nutrient Network standard experimental protocol (58). Nitrogen (as time release urea), phosphorus (as triple super phosphate), and potassium (as K_2_SO_4_) were hand-spread on 5 m × 5 m plots. The application rate of 10 g m^−2^ y^−1^ of each element was chosen because it is expected to exceed plant demand (58). In year 1 and year 10, 100 g of micronutrients were applied with K as Everris Micromax™. Sites had a minimum of three replicates per treatment, but a few sites maintained up to five replicates. In total, the 4 to 14 y of site-level fertilization treatments (*SI Appendix*, Table S1) yielded 15,204 experimental plot-years.

### Biomass and Diversity.

Each site used network protocols to measure peak aboveground live plant biomass (in g m^−2^ y^−1^) and the percent cover of each plant species in designated portions of each plot (58). Biomass was sampled by clipping and drying to constant weight (*SI Appendix, Extended Methods*). Species cover was assessed visually to the nearest percent. From species cover, we derived three diversity metrics: the effective number of species (e^H^), representing species richness if all species were equally abundant (90); Whitaker’s beta (βplot), the ratio of site level species richness to plot-level species richness (91), and Evenness (E), describing the distribution of species relative abundances and the inverse of dominance.

### Site MAP.

For most (59) sites, MAP was derived from daily precipitation measured at a weather station selected by the local investigator (*SI Appendix*, Table S7). MAP was the mean of precipitation summed from harvest to harvest for the selected years of biomass and cover data. For the remaining 12 sites, we determined MAP from downscaled precipitation estimates (92). We validated the comparability of the two sources of MAP (*SI Appendix*, Figs. S5 and S6). We chose site MAP as the predictor of aboveground biomass after screening 31 other potential site metrics of precipitation, temperature, and evaporative demand (*SI Appendix*, Table S8). This analysis did not consider within-site temporal variability in annual precipitation amounts, which has been examined elsewhere (32, 64).

### Statistical Analysis.

Analytic approaches were linear mixed models of the interactive effects of the number or identity of nutrients with MAP across all sites and for subsets of sites assigned to one of four nutrient limitation categories. Community mediation of nutrient and MAP effects on biomass were evaluated using structural equation models. Preparation and analysis of the biomass and diversity data are detailed in the *SI Appendix, Extended Methods*.

## Supplementary Material

Appendix 01 (PDF)

## Data Availability

Datasets and code used in this analysis have been deposited in Dryad (10.5061/dryad.vdncjsz50) (93).
