# Supplementary material for: Interactions among nutrients govern the global grassland biomass–precipitation relationship
Source: Proc Natl Acad Sci U S A. 2025 Apr 11;122(15):e2410748122. doi: 10.1073/pnas.2410748122 (PMC12012460; doi:10.1073/pnas.2410748122)
Supplement: Supplementary file 1 — Appendix 01 (PDF) [file pnas.2410748122.sapp.pdf]

Supporting Information for
Interactions among nutrients govern the global grassland biomass – precipitation relationship.

Philip A. Fay, Laureano A. Gherardi, Laura Yahdjian, Peter B. Adler, Jonathan D. Bakker, Siddharth Bharath, Elizabeth T. Borer, W. Stanley Harpole, Erika Hersch-Green, Travis E. Huxman, Andrew S. MacDougall, Anita C. Risch, Eric W. Seabloom, Sumanta Bagchi, Isabel C. Barrio, Lori Biederman, Yvonne M. Buckley, Miguel N. Bugalho, Maria C. Caldeira, Jane A. Catford, QingQing Chen, Elsa Cleland, Scott L. Collins, Pedro Daleo, Christopher R. Dickman, Ian Donohue, Mary E. DuPre, Nico Eisenhauer, Anu Eskelinen, Nicole Hagenah, Yann Hautier, Robert W. Heckman, Ingibjörg S. Jónsdóttir, Johannes M.H. Knops, Ramesh Laungani, Jason P. Martina, Rebecca L. McCulley, John W. Morgan, Harry Olde Venterink, Pablo L. Peri, Sally A. Power, Xavier Raynaud, Zhengwei Ren, Christiane Roscher, Melinda Smith, Marie Spohn, Carly J. Stevens, Michelle J. Tedder, Risto Virtanen, Glenda M. Wardle, George R. Wheeler

Corresponding Authors:

Philip A. Fay
Email: Philip.fay@usda.gov

Laureano A. Gherardi
Email: lau.gherardi@berkeley.edu

Laura Yahdjian
Email:yahdjian@agro.uba.ar

This PDF file includes:

Extended Methods
Figures S1 to S6
Tables S1 to S8
Climate Variables
SI References

Extended Methods

Data Preparation. We obtained the June 2023 version of the Nutrient Network biomass and plant cover dataset, *comb-by-plot-clim-soil-diversity-2023-06-28*. Preparation of the dataset was performed in SAS 9.4.

Site selection. We selected 71 out of 130 total Nutrient Network sites (Table S1). Sites were included if they conducted the full factorial NPKμ fertilization design for at least four years. We excluded pre-treatment years, sites with only observational data which did not conduct the fertilization experiment, and sites which only conducted the herbivory fence x NPKμ experiment, which does not include the factorial nutrient treatments. The site selection criteria left 15,204 observations available for analysis.

Aboveground biomass calculation. We determined total live aboveground biomass ('aboveground biomass') in g m^{-2} y^{-1} by summing three biomass fractions in the dataset: vascular_live_mass, nonvascular_live_mass, and unsorted_live_mass. Three sites which otherwise met the inclusion criteria did not sort biomass to these fractions in one or more years. For those sites the variable total unsorted_mass (which includes an unquantified amount of dead mass) was assigned to aboveground biomass.

Dataset Repair. Structural errors in the dataset were corrected, mostly deleting occasional treatment years for a site without both aboveground biomass values and total cover and the diversity variables effective species richness (from cover.

Outlier identification. We filtered aboveground biomass for outliers in two steps. First, visual inspection of frequency distribution and Q-Q plots (Proc Univariate) identified four large aboveground biomass values as candidate outliers. Second, we fit a linear mixed model (Proc Mixed) to aboveground biomass as a function of nutrient treatment, year, and their interaction. Cook's D statistic ('influence' option) confirmed that the three largest of the candidate outliers had high influence on model fit and likely represented unusual predictor-response combinations. These three values were set to missing. The frequency distribution of total cover did not indicate any initial outlier candidates so cover and the diversity variables were not subject to further outlier analysis.

Imputation of missing values. After outlier identification, the dataset contained 24 missing values for aboveground biomass and 46 missing values for total cover. Missing values were filled with averages of the available values from the same site, treatment, and year.

Mean Annual Precipitation. See Climate Variables for sources and preparation of mean annual precipitation data.

Graphics. Means and standard errors in original units are used in graphs, which were prepared in OriginPro 10.0.5.157.

Statistical Procedures. The general approach was to apply linear mixed models (Questions 1 and 2) and structural equation models (Question 3).

Linear Models followed the general form:

*aboveground biomass = μ + nutrients + nutrients*MAP + error.* (Equation 1)

Nutrients refers to the N, P, and Kμ fertilization treatments (Question 1), and *MAP* refers to mean annual precipitation, selected after a climate variable screening described below. Equation 1 was implemented in Proc Mixed in SAS STAT version 15.3 coded as:

```
Proc mixed method=reml covtest ic;
Class nutrients site block;
Model aboveground biomass= nutrients * MAP/ddfm=kr;                    (Model 1)
        Random site   block*site    n*p*k*site;
```

Question 1. Does the global biomass-MAP relationship become steeper with increased number of added nutrients?

We applied Model 1 in two ways to test how fertilizing with combinations of N, P, and Kμ affected the biomass-MAP relationship. First, the *nutrients* term was the number of nutrients applied: 0 = Control, 1 for N, P, or Kμ applied singly, 2 for nutrient pairs, and 3 for the NPKμ treatment (Fig. 2A). Second, the *nutrients* term was the factorial combinations: N, P, Kμ, N*P, N*Kμ, P*Kμ, and N*P*Kμ. Terms representing all combinations of nutrient interactions with MAP were also specified. These models provided tests of nutrient main (across-site) effects and nutrient x linear MAP interactions (Figs. 2B, 2C, Table 1). We did not test for non-linear MAP effects in this or subsequent analyses because preliminary analysis revealed no indication of non-linear MAP effects, and because a linear form is the simplest test of the hypotheses. Both forms of Model 1 were also fit to the diversity variables effective species richness (eH), Evenness (E), and plot beta diversity (ßplot) (Fig. S4). For the Question 1 analyses, response variables were averaged at each site by block and fertilizer treatment across years to correspond in temporal scale to site MAP. The response variables and MAP were centered and scaled (mean=0, standard deviation=1).

Question 2. Does the increased steepness of the biomass-MAP relationship correspond to the form of nutrient limitation?

Assignment of Nutrient Limitation Forms to Sites: We assigned each site to one of the forms of nutrient limitation (Table S5) defined in the rubric (Fig. 1). These limitation forms; None, Single, Additive, Sub-additive, and Synergistic; were defined by the site-level response to fertilization with factorial combinations of N and P (Table S6). We did not consider the Kμ fertilization treatment because the analyses for Question 1 indicated that Kμ did not interact with N, P, or MAP to influence aboveground biomass. Negative responses to fertilization are possible and occurred at two sites which were not considered further. The nutrient limitation categories correspond to those in (1), except we combine Harpole's three sub-forms of synergistic colimitation.

Nutrient limitation categories were defined by application of a linear mixed model to aboveground biomass at each site. Because each site was analyzed separately, this analysis used the unstandardized dataset averaged by block, N, P, and treatment year (year_trt), across levels of Kμ.

aboveground biomass = μ + *nutrients* + *nutrients***year_trt* + error. (Equation 2)

where *nutrients* represents N and P main effects and the N x P interaction effect and their interactions with treatment year (Equation 2). Treatment year x nutrient interactions were fit with ar(1) covariance structure (Model 2) to account for this source of variability in site-level aboveground biomass, though nutrient interactions with year were not part of the categorization rubric.

```
proc mixed method=reml covtest ic; by site_code;
class N P site_code block year_trt ;                                    (Model 2)
model aboveground biomass= N P N x P N x year_trt P x year_trt N x P x year_trt/ddfm=kr;
random block;
repeated/subject=block*N*P type=ar(1);
lsmeans N*P/diff=control('0' '0');
```

Categorization was based on two outputs from this mixed model for each site: 1) which nutrient effects (N and P main effects, N x P interaction) were statistically significant defined as $p < 0.10$, and 2) which mean nutrient treatment effects were significantly greater than 0 (lsmeans diff statement, $p < 0.10$) (Table S6).

Next, after sites were assigned to one of the nutrient limitation categories, we refit Model 1 separately to the sites in each category (Figs. 3 and S2). We also conducted a supporting analysis to test whether the biomass-MAP relationships for unfertilized controls differed among the limitation forms with a linear mixed model containing form, MAP, and their interaction (Fig. 4).

For questions 1 and 2, slopes of biomass-MAP relationships were computed from linear regressions fit to site-level treatment means in OriginPro 10.0.5.157.

Question 3. Does the form of nutrient limitation alter mediation of MAP and nutrient effects on biomass by community diversity?

We fit structural equation models using Proc Calis to identify the direct and indirect diversity-mediated effects of the number of nutrients applied (0 = Control, 1 = N or P, 2 = NP) and site MAP on aboveground biomass for sites in each of the four nutrient limitation categories (Fig. 3). The dataset for SEM modeling was aboveground biomass averaged at each site by block and N and P treatments across levels of Kμ and years. Aboveground biomass and MAP were centered and scaled (mean=0, standard deviation=1)

We defined an *a priori* model (Fig. S3) containing paths representing these relationships, including correlations among the diversity variables, which for clarity are not shown. Correlation of 0 was specified between MAP and number of nutrients because nutrient treatment was independent of site MAP.

The *a priori* model was first fit across all sites in all limitation categories. Model fit was evaluated with indices indicating absolute fit (χ^2), parsimony (Root mean square error of approximation, RMSEA), and accounting for sample size (Comparative Fit Index, CFI) following Hooper et al. (2). The *a priori* model proved an adequate fit across all sites (χ^2 = 1.73 p = 0.63; RMSEA = 0, CFI =

0.97), so the model was then fit separately to each nutrient limitation category (Figs. 5 and S3, Table 4).

Supplemental Analysis:

We conducted an additional analysis to examine whether the sites differed by form of nutrient limitation in site level properties (Table S3) related to management, mean annual temperature (MAT), topography, soil texture, or soil total N, P, and K contents. MAT, topography variables, and binary management codes were available for all 68 categorized sites. Soils variables were available for 36 of the sites. The effect of NP limitation on the binary management variables was analyzed with a generalized linear mixed model (Proc GLIMMIX) with a binary link, and with a general linear model (Proc GLM) for the continuous variables.

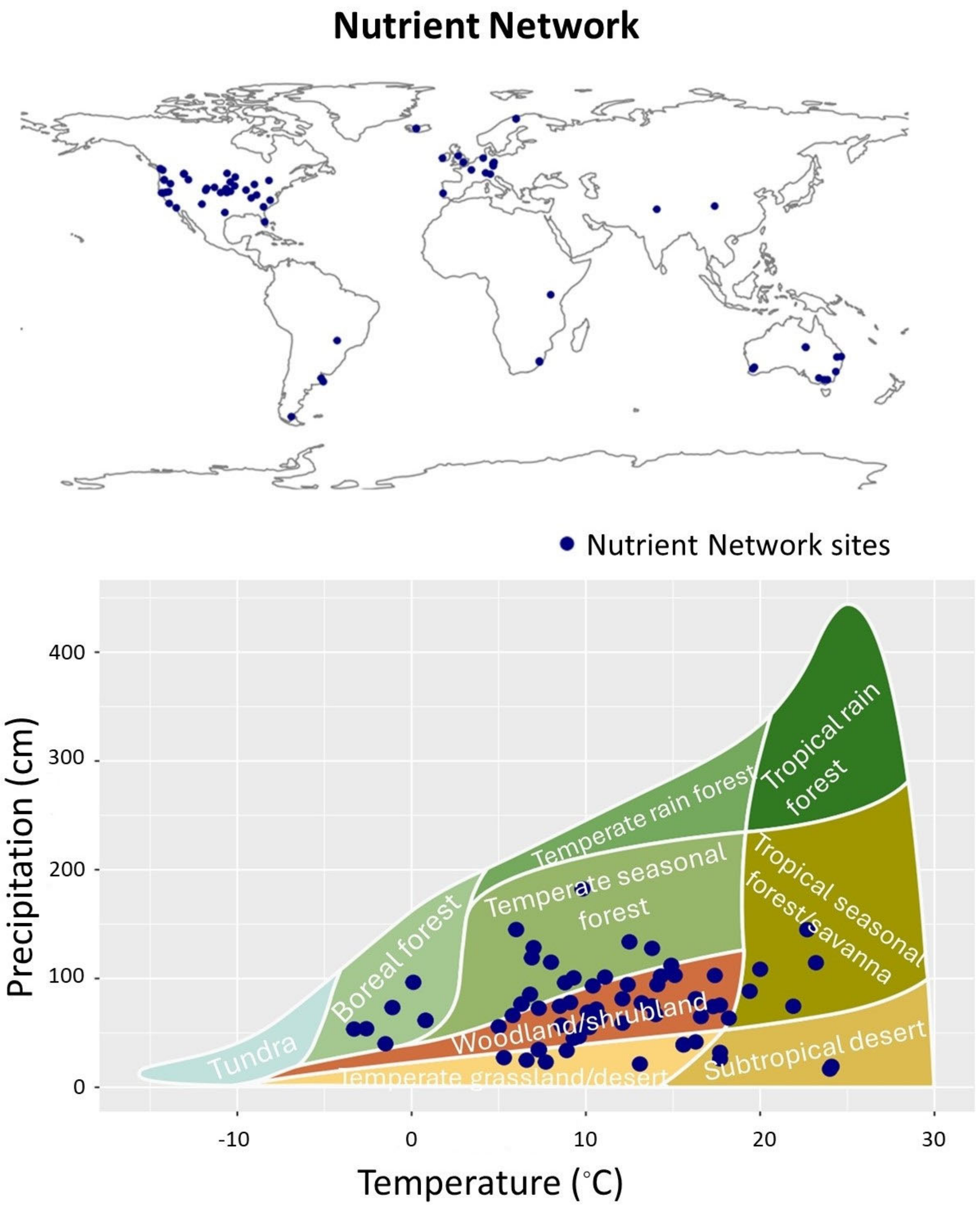

Fig. S1. Upper panel: Geographic extent of 71 grassland sites in the Nutrient Network used to assess how interactions among multiple co-limiting nutrients influence the global grassland biomass-MAP relationship. Lower panel: Distribution of the sites in overlaid on a Whittaker diagram relating mean annual temperature, mean annual precipitation, and the distribution of global biomes plotted in R using George Kunstler's BIOMEplot function (https://github.com/kunstler/BIOMEplot.git).

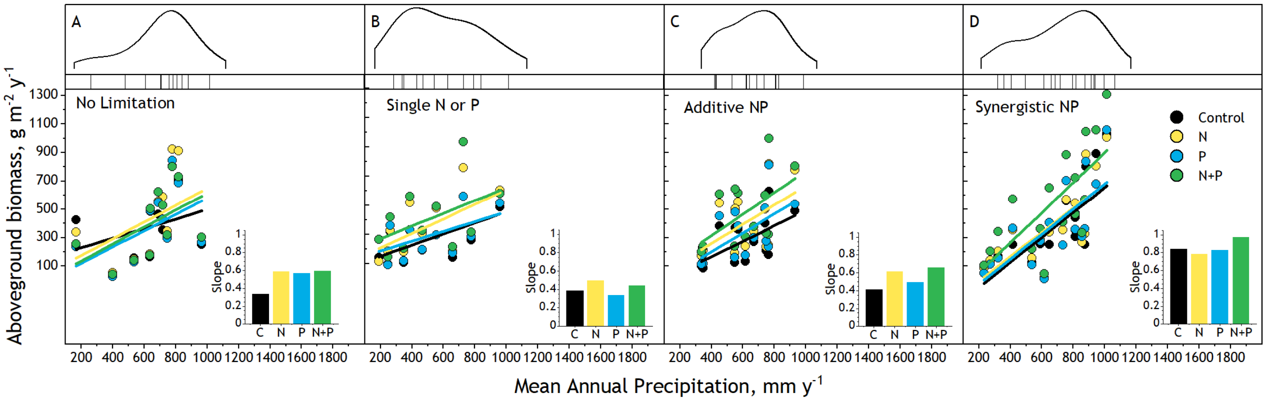

Mixed model effects	No Limitation		Single		Additive		Synergistic	
	F(dfs)	*p*-value	F(dfs)	*p*-value	F(dfs)	*p*-value	F(dfs)	*p*-value
N	6.2(1,204)	0.0137	50.6(1,59)	< 0.0001	232.1(1,112)	< 0.0001	55.0(1,96)	< 0.0001
P	0.6(1,204)	0.4283	1.0(1,59)	0.3125	68.1(1,112)	< 0.0001	48.3(1,96)	< 0.0001
N*P	0.2(1,204)	0.6802	0.2(1,59)	0.6373	0.3(1,112)	0.5797	30.4(1,96)	< 0.0001
MAP	1.1(1,9)	0.3170	7.9(1,9)	0.0208	10.8(1,15)	0.0051	13.3(1,13)	0.0030
MAP*N	3.0(1,204)	0.0835	3.9(1,60)	0.0529	12.4(1,100)	0.0007	1.7(1,93)	0.1917
MAP*P	7.5(1,204)	0.0068	0.7(1,60)	0.4131	3.8(1,100)	0.0525	5.0(1,93)	0.0279
MAP*N*P	1.2(1,204)	0.2742	0.0(1,60)	0.8958	0.2(1,100)	0.6550	4.4(1,93)	0.0397

Fig. S2. Aboveground biomass in relation to mean annual precipitation (MAP) for sites with less than 1013 mm y^{-1} MAP classified by form of response to nitrogen (N) and phosphorus (P) fertilization. N and P treatments are averaged across levels of Kμ fertilization. Insets depict the slopes for unfertilized control (C), N, P, and N together with P. See Fig. 3 for plots containing all sites, and Table S2 for linear regression equations. The table contains linear mixed model analysis of variance F statistics, degrees of freedom (dfs) and *p*-values for tests of the effects of N, P, MAP, and their interactions on aboveground biomass in each class of nutrient limitation.

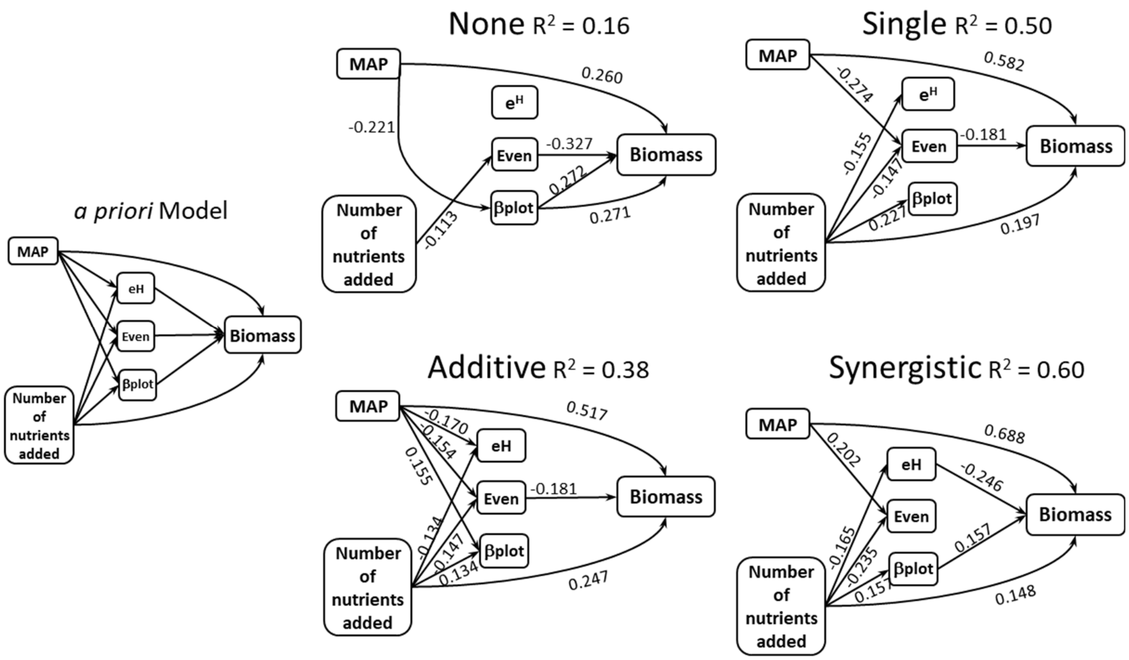

Fig. S3. The *a priori m*odel and the fitted model for each form of nutrient limitation. Only the significant paths are depicted (Table S4) with their standardized coefficients. Indirect effects in Fig. 5C represent community mediation of MAP and nutrient effects on biomass, and combine paths through effective species richness, species evenness, and beta diversity.

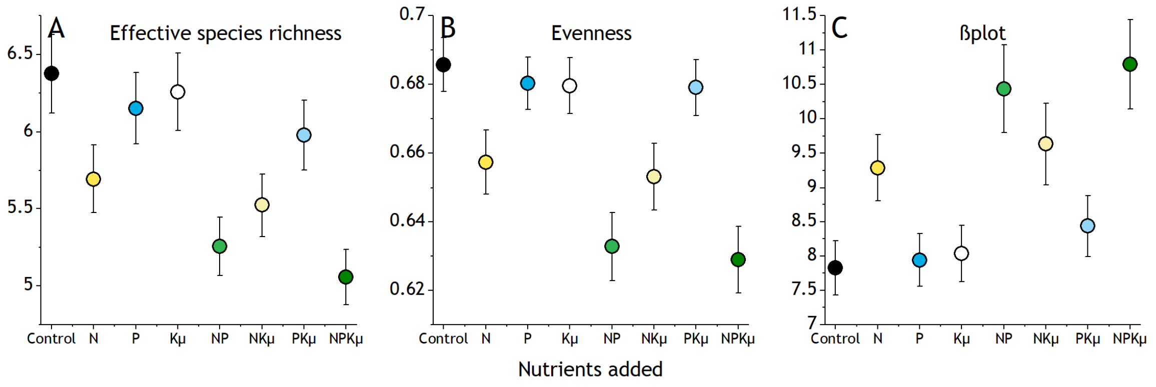

Mixed model effects	eH		Evenness		plotß	
	F(dfs)	*p*-value	F(dfs)	*p*-value	F(dfs)	*p*-value
N	107.3(1,508)	**< 0.0001**	88.0(1,430)	**< 0.0001**	99.0(1,468)	**< 0.0001**
P	21.2(1,508)	**< 0.0001**	11.1(1,430)	**0.0010**	12.5(1,468)	**0.0005**
N*P	1.5(1,508)	0.2217	6.1(1,430)	**0.0138**	4.6(1,468)	**0.0329**
Kµ	4.3(1,508)	**0.0378**	0.8(1,430)	0.3783	2.6(1,468)	0.1072
N*Kµ	0.0(1,508)	0.9496	0.0(1,430)	0.9289	0.0(1,468)	0.9011
P*Kµ	0.1(1,508)	0.7744	0.2(1,430)	0.6691	0.0(1,468)	0.8414
N*P*Kµ	0.0(1,508)	0.9435	0.0(1,430)	0.8313	0.2(1,468)	0.6694
MAP	0.0(1,69)	0.9035	0.3(1,69)	0.5924	0.0(1,70)	0.9821
MAP*N	0.0(1,523)	0.8474	5.3(1,451)	**0.0212**	6.0(1,487)	**0.0149**
MAP*P	8.3(1,523)	**0.0042**	0.3(1,451)	0.5555	0.6(1,487)	0.4543
MAP*N*P	2.0(1,523)	0.1608	0.0(1,451)	0.9486	0.4(1,487)	0.5250
MAP*Kµ	0.4(1,523)	0.5516	0.1(1,451)	0.7883	0.0(1,487)	0.8944
MAP*N*Kµ	2.5(1,523)	0.1112	3.0(1,451)	0.0816	0.5(1,487)	0.4764
MAP*P*Kµ	0.6(1,523)	0.4404	0.3(1,451)	0.5729	1.3(1,487)	0.2639
MAP*N*P*Kµ	0.0(1,523)	0.8995	0.6(1,451)	0.4223	0.3(1,487)	0.5826

Fig. S4. Species diversity metrics (means ± SE) for the eight factorial nutrient treatments averaged across all 71 sites. The table contains linear mixed model analysis of variance F statistics, degrees of freedom (dfs), and *p*-values for tests of the effects of N, P, Kµ, MAP, and their interactions on effective species richness (eH), evenness, and plot beta diversity (ßplot).

Table S1. Nutrient Network sites in this study and their key biophysical properties, sorted from lowest to highest mean annual precipitation (MAP). MAT: mean annual temperature (MAT).

Site code	Site Name(1)	Continent	Latitude, Longitude, °	Elevation, m	MAT, °C	MAP, mm	Unfertilized biomass, g m-2	MAP Source
**ethass.au**	Ethabuka (South Site) (6, G)	Australia	-23.6, 138.4	104	24.0	183	635	Station
**ethamc.au**	Ethabuka (Main Camp) (6, B)	Australia	-23.8, 138.5	104	24.1	192	91	Station
**sevi.us**	Sevilleta LTER (15)	N. Amer.	34.4, -106.7	1600	13.1	212	142	Station
**potrok.ar**	Potrok Aike (6, M)	S. Amer.	-51.9, -70.4	160	6.6	249	88	BIOCLIM
**hart.us**	Hart Mountain (5)	N. Amer.	42.7, -119.5	1508	7.7	255	74	Station
**elliot.us**	Elliott Chaparral (12)	N. Amer.	32.9, -117.1	200	17.7	264	332	Station
**shps.us**	Sheep Experimental Station (5, G)	N. Amer.	44.3, -112.2	1661	5.3	278	138	Station
**mtca.au**	Mt. Caroline (13)	Australia	-31.8, 117.6	285	17.7	317	179	Station
**msla.us**	Missoula (4)	N. Amer.	46.7, -114.0	1169	7.3	326	121	Station
**msla_2.us**	Missoula - MPG Ranch (4)	N. Amer.	46.7, -114.0	1188	7.3	331	103	Station
**msla_3.us**	Missoula - MPG Ranch - 3 (4)	N. Amer.	46.7, -114.0	1158	7.3	331	85	Station
**sgs.us**	Shortgrass Steppe LTER (14)	N. Amer.	40.8, -104.8	1650	8.9	336	103	Station
**kibber.in**	Kibber (Spiti) (5)	Asia	32.3, 78.0	4241	-1.5	400	41	BIOCLIM
**kiny.au**	Kinypanial (10)	Australia	-36.2, 143.8	90	15.6	403	165	Station
**ping.au**	Pingelly Paddock (8, G, C)	Australia	-32.5, 117.0	338	16.3	406	254	Station
**sedg.us**	Sedgwick Reserve UCNRS (10)	N. Amer.	34.7, -120.0	550	15.6	409	356	Station
**badlau.de**	Bad Lauchstaedt (7, M, C)	Europe	51.4, 11.9	120	9.3	451	360	Station
**cdpt.us**	Cedar Point Biological Station (14)	N. Amer.	41.2, -101.6	965	9.6	459	166	Station
**saana.fi**	Saana (8)	Europe	69.0, 20.8	600	-2.6	516	133	Station
**kilp.fi**	KilpisjÃ¤rvi (7, G)	Europe	69.1, 20.9	700	-3.3	544	185	Station
**smith.us**	Smith Prairie (9)	N. Amer.	48.2, -122.6	63	10.2	549	375	Station
**jena.de**	JeNut (9, M)	Europe	50.9, 11.5	320	8.6	556	354	Station
**msum.us**	Minnesota State Univ. Morehead (5, B)	N. Amer.	46.9, -96.5	311	5.0	556	311	BIOCLIM

Table S1, continued.

Site code	Site Name(1)	Continent	Latitude, Longitude, °	Elevation, m	MAT, °C	MAP, mm	Unfertilized biomass, g m-2	MAP Source
**bldr.us**	Boulder South Campus (8, M)	N. Amer.	40.0, -105.2	1633	9.9	562	130	Station
**saline.us**	Saline Experimental Range (8)	N. Amer.	39.1, -99.1	555	12.1	590	244	Station
**ahth.is**	Audkuluheidi Heath (4, G)	Europe	65.1, -19.7	470	0.8	615	159	BIOCLIM
**amlr.is**	Audkuluheidi Melur (4, G)	Europe	65.1, -19.7	470	0.8	615	12	BIOCLIM
**burrawan.au**	Burrawan (11)	Australia	-27.7, 151.1	425	18.2	627	195	Station
**cereep.fr**	CEREEP - Ecotron IDF (8, G, C)	Europe	48.3, 2.7	83	10.8	631	519	CRU
**comp.pt**	Companhia das Lezirias (9, G, C)	Europe	38.8, -8.8	20	16.6	679	262	Station
**sage.us**	Sagehen Creek UCNRS (6)	N. Amer.	39.4, -120.2	1920	5.8	688	127	Station
**mcla.us**	Mclaughlin UCNRS (13)	N. Amer.	38.9, -122.4	642	14.0	689	270	Station
**hero.uk**	Heronsbrook (Silwood Park) (5)	Europe	51.4, -0.6	60	10.2	690	509	Station
**rook.uk**	Rookery (Silwood Park) (5)	Europe	51.4, -0.6	60	10.1	690	180	Station
**lake.us**	Lakeside Laboratory (4, B, C)	N. Amer.	43.4, -95.2	452	7.3	726	611	BIOCLIM
**azitwo.cn**	Azi Two (4)	Asia	33.6, 101.5	3500	-1.1	733	247	BIOCLIM
**bayr.de**	Bayreuth (5, M, C)	Europe	49.9, 11.6	340	8.5	745	140	BIOCLIM
**sereng.tz**	Serengeti (4)	Africa	-2.4, 34.9	1536	21.9	746	273	CRU
**pape.de**	Papenburg (6, C)	Europe	53.1, 7.5	0	9.1	748	930	CRU
**ukul.za**	Ukulinga (11, M)	Africa	-29.7, 30.4	842	17.7	769	548	Station
**cdcr.us**	Cedar Creek LTER (14)	N. Amer.	45.4, -93.2	270	6.3	771	175	Station
**nilla.au**	Nillahcootie (6, G, C)	Australia	-36.9, 146.0	280	13.8	776	159	Station
**koffler.ca**	Koffler Reserve (11, M)	N. Amer.	44.0, -79.5	301	6.3	778	654	Station
**yarra.au**	Yarramundi (7, M, C)	Australia	-33.6, 150.7	19	17.3	779	417	Station
**hopl.us**	Hopland REC (13)	N. Amer.	39.0, -123.1	598	13.2	792	242	Station
**konz.us**	Konza LTER (11, B)	N. Amer.	39.1, -96.6	440	12.1	808	463	Station
**sier.us**	Sierra Foothills REC (13)	N. Amer.	39.2, -121.3	197	16.3	826	313	Station
**temple.us**	USDA ARS Temple, TX (11)	N. Amer.	31.0, -97.4	184	19.4	836	843	Station

(1) Number of years of aboveground biomass data analyzed. B: burned, C: cultivated grassland, G: grazed, M: unspecified management.

Table S1, continued.

Site code	Site Name(1)	Continent	Latitude, Longitude, °	Elevation, m	MAT, °C	MAP, mm	Unfertilized biomass, g m-2	MAP Source
**bnch.us**	Bunchgrass (Andrews LTER) (14)	N. Amer.	44.3, -122.0	1318	6.8	858	278	CRU
**doane.us**	Doane College (7, B, C)	N. Amer.	40.7, -96.9	418	10.6	890	376	Station
**cowi.ca**	Cowichan (14)	N. Amer.	48.8, -123.6	50	10.4	916	485	Station
**gilb.za**	Mt Gilboa (4, B)	Africa	-29.3, 30.3	1748	14.1	943	242	BIOCLIM
**bnbt.us**	Benedictine Bottoms (5, B)	N. Amer.	39.6, -95.1	240	12.4	944	976	BIOCLIM
**kbs.us**	Kellogg Biological Station LTER (6)	N. Amer.	42.4, -85.4	288	8.8	954	539	Station
**valm.ch**	Val Mustair (12)	Europe	46.6, 10.4	2320	0.1	989	211	Station
**trel.us**	Trelease (9)	N. Amer.	40.1, -88.8	200	11.1	994	991	Station
**cbgb.us**	Chichaqua Bottoms (12, B, C)	N. Amer.	41.8, -93.4	274	9.3	999	434	Station
**chilcas.ar**	Las Chilcas (9)	S. Amer.	-36.3, -58.3	15	15.1	1003	610	Station
**marc.ar**	Mar Chiquita (11)	S. Amer.	-37.7, -57.4	6	14.3	1038	739	Station
**sava.us**	Savannah River (5)	N. Amer.	33.3, -81.7	71	17.4	1061	92	Station
**pinj.au**	Pinjarra Hills (4, G, C)	Australia	-27.5, 152.9	38	20.0	1085	777	BIOCLIM
**lagoas.br**	Tres Lagoas (7, B, C)	S. Amer.	-21.0, -51.8	279	23.2	1145	240	BIOCLIM
**lancaster.uk**	Lancaster (9, G)	Europe	54.0, -2.6	202	8.0	1147	104	Station
**unc.us**	Duke Forest (4, C)	N. Amer.	36.0, -79.0	141	14.9	1153	351	Station
**look.us**	Lookout (Andrews LTER) (14)	N. Amer.	44.2, -122.1	1500	6.9	1190	244	CRU
**frue.ch**	Fruebuel (7, G, C)	Europe	47.1, 8.5	995	7.0	1287	794	Station
**hall.us**	Hall's Prairie (7, M)	N. Amer.	36.9, -86.7	194	13.8	1322	528	Station
**spin.us**	Spindletop (14, G, C)	N. Amer.	38.1, -84.5	271	12.5	1356	456	Station
**arch.us**	Archbold Biological Station (7, B)	N. Amer.	27.2, -81.2	8	22.7	1420	403	Station
**bogong.au**	Bogong (14)	Australia	-36.9, 147.3	1760	6.0	1488	539	Station
**burren.ie**	Slieve Carran (5, G)	Europe	53.1, -9.0	104	9.8	1839	511	Station

(1) Number of years of aboveground biomass data analyzed. B: burned, C: cultivated grassland, G: grazed, M: unspecified management.

Table S2. Equations and coefficients of determination (adjusted R^2) for linear regressions relating aboveground biomass to mean annual precipitation (MAP) in Figs. 2 - 4, and Fig. S2. Equation form: Aboveground biomass (AGB) = Slope(SE) * MAP + Intercept(SE). Bolded slopes are significant $p < 0.05$. † $p < 0.10$.

All Sites and Limitation Forms - Fig. 2			
**Number of Nutrients Added**		**Factorial Treatments**	
0	AGB = **0.312**(0.074) * MAP + 112.2(60.4) R^2 = 0.19	Control	AGB = **0.310**(0.073) * MAP + 106.1(59.8) R^2 = 0.21
1	AGB = **0.372**(0.069) * MAP + 96.5(56.4) R^2 = 0.29	N	AGB = **0.391**(0.083) * MAP + 122.0(68.2) R^2 = 0.25
2	AGB = **0.416**(0.077) * MAP + 121.3(62.7) R^2 = 0.29	P	AGB = **0.368**(0.069) * MAP + 86.6(56.6) R^2 = 0.30
3	AGB = **0.470**(0.094) * MAP + 138.8(76.3) R^2 = 0.26	K	AGB = **0.355**(0.064) * MAP + 67.4(52.3) R^2 = 0.32
		NP	AGB = **0.422**(0.091) * MAP + 167.1(74.6) R^2 = 0.25
		NK	AGB = **0.430**(0.077) * MAP + 97.3(63.1) R^2 = 0.32
		PK	AGB = **0.376**(0.072) * MAP + 93.5(58.8) R^2 = 0.29
		NPK	AGB = **0.466**(0.096) * MAP + 140.4(78.5) R^2 = 0.26

	All MAP - Fig. 3	Sites < 1013 MAP Fig. S2
**No limitation**		
Control	AGB = -0.020(0.268) * MAP + 357.1(202.4) R^2 = -0.09	AGB = 0.337(0.341) * MAP + 161.1(231.2) R^2 = 0.00
N	AGB = 0.055(0.337) * MAP + 349.6(254.8) R^2 = -0.09	AGB = 0.591(0.402) * MAP + 56.3(271.9) R^2 = 0.10
P	AGB = 0.108(0.284) * MAP + 260.1(214.4) R^2 = -0.08	AGB = 0.572(0.335) * MAP + 5.9(226.6) R^2 = 0.16
NP	AGB = 0.113(0.285) * MAP + 282.3(215.6) R^2 = -0.08	AGB = 0.596(0.331) * MAP + 17.5(223.9) R^2 = 0.18
**Single N or P**		
Control	AGB = **0.274**(0.087) * MAP + 130.1(76.2) R^2 = 0.36	AGB = **0.385**(0.160) * MAP + 73.2(89.4) R^2 = 0.33
N	AGB = **0.338**(0.112) * MAP + 182.7(97.8) R^2 = 0.34	AGB = **0.498**(0.218) * MAP + 109.5(121.9) R^2 = 0.30
P	AGB =**0.275**(0.085) * MAP + 156.6(74.3) R^2 = 0.37	AGB =**0.339†**(0.164) * MAP + 118.8(92.0) R^2 = 0.25
NP	AGB = **0.324**(0.120) * MAP + 240.0(105.4) R^2 = 0.28	AGB = 0.439(0.267) * MAP + 183.9(149.8) R^2 = 0.14
**Additive NP**		
Control	AGB = **0.348**(0.105) * MAP + 70.1(91.8) R^2 = 0.36	AGB = **0.547**(0.210) * MAP + - 53.5(131.1) R^2 = 0.31
N	AGB = **0.404**(0.147) * MAP + 160.7(128.9) R^2 = 0.27	AGB = **0.680**(0.289) * MAP + - 13.2(180.5) R^2 = 0.26
P	AGB = **0.401**(0.123) * MAP + 96.1(107.5) R^2 = 0.35	AGB = **0.663**(0.258) * MAP + - 63.7(161.3) R^2 = 0.30
NP	AGB = **0.460**(0.154) * MAP + 194.2(135.1) R^2 = 0.30	AGB = **0.759**(0.319) * MAP + 10.3(199.4) R^2 = 0.26
**Synergistic NP**		
Control	AGB = **0.879**(0.217) * MAP + -225.0(153.0) R^2 = 0.49	AGB = **0.879**(0.217) * MAP + -225.0(153.0) R^2 = 0.49
N	AGB = **0.854**(0.207) * MAP + -174.3(146.3) R^2 = 0.50	AGB = **0.854**(0.207) * MAP + -174.3(146.3) R^2 = 0.50
P	AGB = **0.882**(0.211) * MAP + -204.2(148.8) R^2 = 0.51	AGB = **0.882**(0.211) * MAP + -204.2(148.8) R^2 = 0.51
NP	AGB = **1.058**(0.271) * MAP + -158.2(190.9) R^2 = 0.47	AGB = **1.058**(0.271) * MAP + -158.2(190.9) R^2 = 0.47

Table S3. Means and standard errors (SE) of site characteristics, management, mean annual temperature (MAT), geographic position, and soil properties for sites categorized by form of nutrient limitation. F and *p*-value are for tests for differences between limitation forms.

Site Characteristic	F	*p*-value	No Limitation		Single		Additive		Synergistic	
			Mean	SE	Mean	SE	Mean	SE	Mean	SE
Anthropogenic	0.89	0.4489	0.25	0.13	0.21	0.10	0.35	0.11	0.12	0.08
Burned	1.28	0.2874	0.00	0.00	0.32	0.11	0.05	0.05	0.18	0.10
Grazed	1.16	0.3329	0.42	0.15	0.16	0.09	0.15	0.08	0.24	0.11
Managed	0.70	0.5583	0.25	0.13	0.37	0.11	0.50	0.11	0.35	0.12
MAT	0.30	0.8260	11.26	2.68	12.21	1.41	10.38	0.73	10.75	1.67
Latitude	0.87	0.4625	39.12	5.17	38.32	1.82	43.54	1.32	40.54	2.70
Elevation	0.72	0.5420	815.00	374.33	560.68	132.09	503.45	125.00	829.76	214.62
% N	0.39	0.7622	0.36	0.12	0.23	0.04	0.30	0.08	0.34	0.09
% K	0.13	0.9410	204.56	60.52	236.15	32.59	195.24	55.48	224.31	56.89
% P	1.85	0.1506	69.89	18.38	46.08	16.66	48.06	8.02	26.08	5.37
% Clay	0.54	0.6554	15.50	3.88	14.21	2.21	12.28	2.41	17.96	4.95
% Sand	1.15	0.3434	62.36	9.15	58.47	6.58	54.74	7.31	42.41	7.64
% Silt	1.51	0.2309	22.04	5.75	27.26	4.90	32.91	5.59	39.57	5.41

Table S4. Standardized direct, indirect, and total effects of mean annual precipitation (MAP), number of nutrients added (# Nutrients), effective species richness (eH), evenness, and plot beta diversity (*β*plot) on aboveground biomass for all sites and for sites categorized by form of limitation by nitrogen and phosphorus (Figs. 5, S3).

Kind of Limitation	Predictor	Standardized Effects on Aboveground Biomass					
		**Direct**	***p*-value**	**Indirect**	***p*-value**	**Total**	***p*-value**
All	MAP	0.510	< 0.0001	0.015	0.0350	0.525	< 0.0001
	# Nutrients	0.170	< 0.0001	0.031	< 0.0001	0.200	< 0.0001
	eH	-0.122	< 0.0001	- -	- -	-0.122	< 0.0001
	Evenness	- -	- -	- -	- -	- -	- -
	ßplot	0.120	< 0.0001	- -	- -	0.120	< 0.0001
No Limitation	MAP	0.260	0.0001	-0.076	0.0799	0.184	0.0148
	# Nutrients	-0.032	0.6297	0.032	0.1110	0.000	0.9965
	eH	0.073	0.3827	- -	- -	0.073	0.3827
	Evenness	-0.327	0.0008	- -	- -	-0.327	0.0008
	ßplot	0.272	0.0021	- -	- -	0.272	0.0021
Single	MAP	0.583	< 0.0001	0.049	0.0237	0.631	< 0.0001
	# Nutrients	0.197	< 0.0001	0.048	0.0121	0.245	< 0.0001
	eH	- -	- -	- -	- -	- -	- -
	Evenness	-0.181	0.0013	- -	- -	-0.181	0.0013
	ßplot	0.092	0.0930	- -	- -	0.092	0.0930
Additive	MAP	0.517	< 0.0001	0.025	0.0443	0.542	< 0.0001
	# Nutrients	0.248	< 0.0001	0.024	0.0448	0.272	< 0.0001
	eH	- -	- -	- -	- -	- -	- -
	Evenness	-0.181	0.0016	- -	- -	-0.181	0.0016
	ßplot	-0.018	0.7554	- -	- -	-0.018	0.7554
Synergistic	MAP	0.688	< 0.0001	-0.021	0.4083	0.667	< 0.0001
	# Nutrients	0.149	0.0010	0.065	0.0104	0.214	< 0.0001
	eH	-0.246	< 0.0001	- -	- -	-0.246	< 0.0001
	Evenness	- -	- -	- -	- -	- -	- -
	ßplot	0.157	0.0021	- -	- -	0.157	0.0021

Table S5. Rubric to apply the conceptual framework of Fig. 1 to identify the form of nutrient limitation expressed at each site from responses to fertilization with nitrogen (N), phosphorus (P) or both (NP), relative to unfertilized controls. Subadditive and synergistic co-limitation can occur with different patterns of individual nutrient responses.

<table>
<tr><th colspan="2">Mixed Model Effects</th><th>Relationships among Means</th><th colspan="3">Patterns of response to fertilization with N, P, and NP.</th></tr>
<tr><td rowspan="6">Additive Forms:
N x P interaction: $p > 0.10$
N + P = NP</td><td></td><td></td><td colspan="3">No Limitation by N or P</td></tr>
<tr><td>No main effects for N or P ($p > 0.10$)</td><td>N, P, NP = 0</td><td>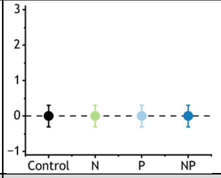
</td><td></td><td></td></tr>
<tr><td></td><td></td><td colspan="3">Single Limitation by N or by P</td></tr>
<tr><td>Significant main effect for one nutrient ($p <$ 0.10) and not the other.</td><td>$\overline{X}$ (N, NP) > 0
$\overline{X}$ (Control, P) = 0
-or-
$\overline{X}$ (P, NP) > 0
$\overline{X}$ (Control, N) = 0</td><td>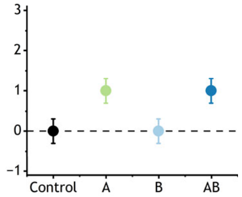
</td><td>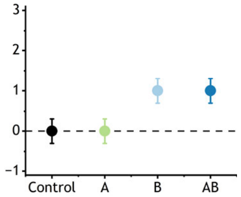
</td><td></td></tr>
<tr><td></td><td></td><td colspan="3">Additive co-limitation by N & P</td></tr>
<tr><td>Significant main effect for N and P ($p < 0.10$)</td><td>$\overline{X}$(N, NP) > 0
$\overline{X}$(P, NP) > 0
$\overline{X}$(N, P) $<$ NP</td><td>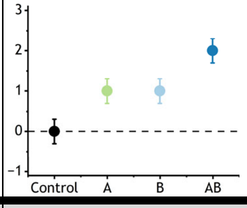
</td><td></td><td></td></tr>
<tr><td rowspan="4">Non-Additive Forms:
N x P interaction $p < 0.10$
N + P ≠ NP</td><td></td><td></td><td colspan="3">Subadditive co-limitation by N & P</td></tr>
<tr><td>None, one (N or P) or two (N and P) significant main effects ($p < 0.10$)</td><td>N and/or P > 0
NP ≥ 0
NP ≤ (N+P)</td><td>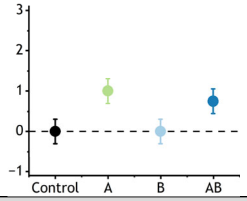
</td><td>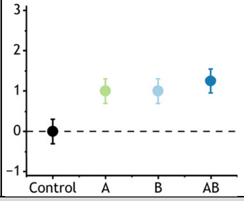
</td><td></td></tr>
<tr><td></td><td></td><td colspan="3">Synergistic co-limitation by N & P</td></tr>
<tr><td>None, one (N or P) or two (N and P) significant main effects ($p < 0.10$)</td><td>N and/or P ≥ 0
NP > 0
NP $>$ (N+P)</td><td>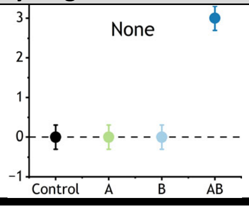
</td><td>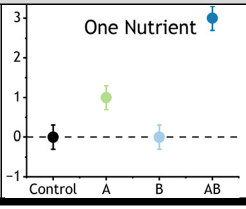
</td><td>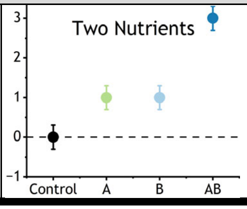
</td></tr>
</table>

Table S6. Statistics from linear mixed models analysis of variance used to assign sites to categories of single nutrient limitation and multiple nutrient co-limitation of aboveground biomass production by nitrogen (N) and phosphorus (P). Effects are least squared mean differences from controls, shown in bold when $p < 0.10$.

	N & P Main Effects				NxP Interaction	Single Treatment Effects					
**Site**	N	*p*-value	P	*p*-value	*p*-value	N	*p*-value	P	*p*-value	NP	*p*-value
**Synergistic (n = 15)**											
amlr.is	**189.0**	0.0003	**65.0**	0.0360	0.0069	51.5	0.3241	-28.6	0.5798	**243.7**	0.0002
comp.pt	**49.2**	0.0000	**79.8**	0.0000	0.0291	32.7	0.1054	**61.3**	0.0035	**157.1**	<0.0001
ping.au	**50.0**	0.0000	**55.3**	0.0000	0.0467	**40.3**	0.0081	**45.4**	0.0032	**127.7**	<0.0001
gilb.za	**45.0**	0.0000	**61.6**	0.0000	0.0066	**29.6**	0.0226	**45.1**	0.0012	**125.9**	<0.0001
mtca.au	**68.5**	0.0000	**34.1**	0.0000	0.0000	**23.5**	0.0475	-5.2	0.6557	**104.8**	<0.0001
saana.fi	**60.6**	0.0000	**18.4**	0.0198	0.0008	**26.8**	0.0331	-9.9	0.4224	**78.5**	<0.0001
azitwo.cn	**53.5**	0.0000	**9.0**	0.0176	0.0175	**42.6**	<0.0001	0.0	0.9988	**64.4**	<0.0001
konz.us	**38.1**	0.0000	**21.2**	0.0000	0.0013	**22.6**	0.0022	6.8	0.3387	**63.0**	<0.0001
shps.us	**44.0**	0.0001	**18.3**	0.0475	0.0027	11.7	0.3903	-10.7	0.4320	**61.0**	<0.0001
ukul.za	**15.9**	0.0530	**40.5**	0.0000	0.0944	2.5	0.8464	**25.5**	0.0519	**58.9**	<0.0001
saline.us	**22.1**	0.0005	**17.8**	0.0038	0.0869	12.2	0.1692	8.0	0.3632	**41.9**	<0.0001
trel.us	**10.8**	0.0471	**16.0**	0.0049	0.0180	-2.2	0.7755	2.6	0.7308	**26.8**	0.0009
bnbt.us	**20.3**	0.0829	1.0	0.9248	0.0189	-6.8	0.6397	-23.7	0.1001	18.8	0.1879
hart.us	30.3	0.1711	-14.2	0.4215	0.0464	-11.1	0.6347	**-47.6**	0.0498	9.7	0.6803
bnch.us	-2.6	0.6360	**11.3**	0.0592	0.0446	**-13.8**	0.0790	-0.7	0.9285	7.9	0.3118
**Additive (n = 21)**											
msla_3.us	**123.4**	0.0000	**19.7**	0.0587	0.9289	**147.0**	<0.0001	35.8	0.1585	**179.6**	<0.0001
bayr.de	**75.9**	0.0000	**30.3**	0.0010	0.3710	**77.7**	<0.0001	**31.8**	0.0564	**129.9**	<0.0001
sgs.us	**75.5**	0.0000	**15.9**	0.0102	0.2301	**70.0**	<0.0001	11.5	0.3205	**101.2**	<0.0001
msla.us	**61.4**	0.0000	**23.7**	0.0290	0.6877	**63.6**	0.0021	25.7	0.1991	**100.6**	<0.0001
bldr.us	**51.6**	0.0013	**27.2**	0.0442	0.7518	**53.9**	0.0275	29.3	0.2141	**93.6**	0.0004
cbgb.us	**60.8**	0.0000	**13.7**	0.0233	0.8408	**68.5**	<0.0001	**20.1**	0.0800	**85.3**	<0.0001

Table S6, continued.

	**N & P Main Effects**				**NxP Interaction**	**Single Treatment Effects**					
**Site**	N	*p*-value	P	*p*-value	*p*-value	N	*p*-value	P	*p*-value	NP	*p*-value
hall.us	**61.2**	0.0000	**16.4**	0.0438	0.2961	**53.7**	0.0004	10.1	0.4811	**85.1**	<0.0001
cdcr.us	**35.5**	0.0000	**31.1**	0.0000	0.3143	**35.6**	<0.0001	**31.1**	<0.0001	**77.6**	<0.0001
smith.us	**34.8**	0.0000	**28.0**	0.0000	0.5691	**36.2**	0.0002	**29.4**	0.0020	**72.9**	<0.0001
cowi.ca	**54.4**	0.0000	**6.2**	0.0961	0.6762	**59.1**	<0.0001	10.0	0.1409	**65.2**	<0.0001
nilla.au	**19.0**	0.0812	**39.3**	0.0023	0.5637	15.2	0.3896	**35.1**	0.0549	**64.7**	0.0011
koffler.ca	**26.4**	0.0000	**24.9**	0.0000	0.9183	**31.0**	0.0012	**29.5**	0.0019	**59.1**	<0.0001
badlau.de	**38.1**	0.0000	**14.3**	0.0203	0.9890	**41.6**	0.0002	**17.4**	0.0936	**58.8**	<0.0001
look.us	**30.7**	0.0006	**16.1**	0.0491	0.9312	**34.6**	0.0112	19.6	0.1436	**52.6**	0.0002
sier.us	**23.3**	0.0010	**25.1**	0.0004	0.1881	15.4	0.1464	17.2	0.1065	**52.3**	<0.0001
yarra.au	**20.6**	0.0057	**22.8**	0.0025	0.9294	**24.0**	0.0405	**26.3**	0.0252	**48.9**	<0.0001
burren.ie	**13.5**	0.0453	**26.8**	0.0005	0.5561	10.8	0.3011	**23.9**	0.0265	**43.4**	0.0002
chilcas.ar	**21.7**	0.0039	**13.8**	0.0520	0.3968	**31.0**	0.0083	**22.7**	0.0495	**40.0**	0.0008
mcla.us	**27.1**	0.0000	**9.1**	0.0939	0.8948	**27.6**	0.0020	9.6	0.2730	**38.8**	<0.0001
frue.ch	**28.0**	0.0000	**7.5**	0.0716	0.5333	**32.6**	<0.0001	**11.7**	0.0872	**38.4**	<0.0001
temple.us	**18.5**	0.0001	**11.7**	0.0086	0.1009	**11.3**	0.0827	4.8	0.4625	**31.2**	<0.0001
					**Single N (n = 13)**						
msla_2.us	**67.7**	0.0000	11.5	0.2870	0.6958	**65.4**	0.0020	9.7	0.6313	**86.3**	<0.0001
sedg.us	**64.1**	0.0000	5.3	0.2222	0.2734	**58.1**	<0.0001	0.7	0.9283	**71.2**	<0.0001
jena.de	**56.6**	0.0000	9.2	0.1657	0.5340	**53.2**	<0.0001	6.5	0.5801	**70.0**	<0.0001
cdpt.us	**61.8**	0.0000	4.9	0.2555	0.4584	**58.4**	<0.0001	2.2	0.7788	**68.8**	<0.0001
lake.us	**51.7**	0.0569	13.4	0.5368	0.5576	36.8	0.3145	0.8	0.9821	**67.8**	0.0750
msum.us	**63.1**	0.0000	1.8	0.8151	0.8406	**61.2**	0.0001	0.3	0.9815	**65.5**	<0.0001
bogong.au	**51.4**	0.0000	4.3	0.2735	0.7139	**55.1**	<0.0001	7.4	0.3015	**58.8**	<0.0001
potrok.ar	**68.1**	0.0589	-5.9	0.8059	0.7678	53.4	0.2314	-16.6	0.7025	55.0	0.2191
spin.us	**24.6**	0.0000	6.1	0.1524	0.4547	**21.5**	0.0016	3.2	0.6260	**31.8**	<0.0001

Table S6, continued.

	N & P Main Effects				NxP Interaction	Single Treatment Effects					
**Site**	N	*p*-value	P	*p*-value	*p*-value	N	*p*-value	P	*p*-value	NP	*p*-value
arch.us	**24.3**	0.0058	-2.3	0.7559	0.9358	**24.8**	0.0400	-1.9	0.8705	**21.5**	0.0724
hopl.us	**15.6**	0.0021	3.2	0.4919	0.4498	**20.0**	0.0066	7.4	0.3084	**19.7**	0.0076
kbs.us	**18.1**	0.0009	0.0	0.9979	0.3493	**23.6**	0.0026	5.0	0.5086	**18.6**	0.0167
marc.ar	**14.8**	0.0191	-1.3	0.8273	0.6248	11.5	0.1818	-4.3	0.6171	13.2	0.1278
**Single P (n = 3)**											
ethamc.au	-6.6	0.8530	**103.4**	0.0711	0.8103	-21.2	0.7637	80.5	0.2576	83.3	0.2422
unc.us	16.4	0.2174	**40.2**	0.0101	0.8228	24.0	0.3015	**48.6**	0.0431	**65.4**	0.0085
elliot.us	9.6	0.1107	**20.4**	0.0015	0.2250	2.5	0.7840	12.9	0.1519	**30.8**	0.0009
**Not N or P (n = 15)**											
doane.us	45.7	0.1176	2.2	0.9200	0.5180	70.2	0.1247	22.4	0.5962	53.7	0.2238
sage.us	26.9	0.1832	20.3	0.2967	0.8642	25.8	0.4029	19.2	0.5318	**52.3**	0.0993
lagoas.br	13.5	0.4185	22.4	0.2033	0.5266	3.1	0.8998	11.5	0.6439	37.0	0.1443
hero.uk	15.8	0.1837	14.3	0.2235	0.8905	18.9	0.2970	17.4	0.3360	**32.8**	0.0762
valm.ch	10.0	0.1707	9.9	0.1763	0.6067	6.5	0.5407	6.3	0.5491	**20.5**	0.0563
kibber.in	46.8	0.2739	-25.7	0.3935	0.9608	41.7	0.4169	-29.2	0.5680	8.9	0.8614
sava.us	-7.4	0.7420	18.6	0.4689	0.2383	-32.6	0.2863	-10.1	0.7383	8.5	0.7776
burrawan.au	6.7	0.4653	0.2	0.9837	0.6352	11.4	0.3948	4.7	0.7263	7.1	0.5952
cereep.fr	2.2	0.6469	2.3	0.6402	0.8529	1.3	0.8469	1.4	0.8417	4.6	0.5133
sereng.tz	9.9	0.3125	-6.9	0.4420	0.8591	11.3	0.4004	-5.6	0.6743	2.3	0.8617
pinj.au	16.8	0.2374	-12.9	0.2914	0.4056	28.1	0.1579	-3.2	0.8715	1.8	0.9264
lancaster.uk	3.5	0.7606	-1.9	0.8646	0.6738	-1.3	0.9329	-6.6	0.6759	1.5	0.9244
pape.de	4.8	0.7225	-4.7	0.7158	0.5544	13.0	0.5069	3.2	0.8684	-0.1	0.9948
kilp.fi	2.4	0.8039	-12.5	0.1635	0.3921	-5.3	0.6663	-19.6	0.1135	-10.0	0.4142
ethass.au	-9.8	0.7219	-36.1	0.1342	0.5812	-19.5	0.5215	-44.4	0.1483	-40.2	0.1896

Table S6, continued.

	N & P Main Effects				NxP Interaction	Single Treatment Effects					
**Site**	N	*p*-value	P	*p*-value	*p*-value	N	*p*-value	P	*p*-value	NP	*p*-value
**Subadditive (n = 2)**											
kiny.au	6.2	0.4290	**23.1**	0.0082	0.0677	**25.3**	0.0651	**43.7**	0.0019	**33.6**	0.0153
sevi.us	-2.0	0.8185	11.6	0.2413	0.0642	19.0	0.2165	**34.1**	0.0418	10.3	0.4891
**Negative (n = 2)**											
rook.uk	**-21.1**	0.0160	**25.8**	0.0191	0.0021	9.0	0.5603	**63.7**	0.0003	-0.8	0.9561
ahth.is	8.4	0.4573	**-28.8**	0.0047	0.1492	23.0	0.1257	-16.8	0.2562	-24.3	0.1056

Climate Variables

Assembly and Validation of Site Mean Annual Precipitation. We assembled a precipitation dataset containing the mean annual precipitation (MAP) and mean annual potential evapotranspiration (MPET) for each site across the years when biomass and cover data were sampled. These and other variables were subsequently screened for use in analyses of nutrient – precipitation interactions.

The primary source was measured monthly precipitation and air temperature from weather stations in the Global Historic Climatology Network (GHCN) database. We chose weather stations confirmed by site PIs to suitably represent the climate of their sites. Monthly PET was computed using the Hargreaves method (3). Annual precipitation and potential evapotranspiration (PET) were summed on a harvest-year basis from the month following the previous harvest to the month of the current harvest. This dataset was an updated version of the dataset compiled by Bharath et al. (4). Site MAP and MPET based on these data are denoted 'Station' in Table S7.

Some sites and years were not present in the GHCN database. For these, we used calendar year annual precipitation and PET from the CRU TS v 4.1 dataset (Climatic Research Unit, University of East Anglia, and NCAS https://crudata.uea.ac.uk/cru/data/hrg/), compiled by Siddharth Bharath. CRU annual precipitation values are determined eon a 0.5° x 0.5° grid derived from observed weather. Sites where MAP and mPET are partially or wholly derived from CRU data are denoted 'CRU' in Table S7.

Station data gapfilled with and CRU data yielded annual precipitation and PET values for 59 of the 71 selected sites (Table S7). We checked the comparability of CRU and Station values by plotting CRU against Station for these 59 sites (Figure S5). For most sites CRU values were highly correlated ($R^2 > 0.96$) and unbiased (slopes near 1.0) representations of Station values for precipitation and PET. For a few sites (magenta data points) CRU either overestimated (cowi.ca, valm.ch, smith.us, which are in topographically complex areas) or underestimated (bnch.us, look.us) Station values. In each case we retained the source with the lower values.

For 12 remaining sites without Station or CRU data we used long-term MAP and mPET derived from the *BIOCLIM* database (5) which was available in the Nutrient Network biomass/diversity dataset for all sites. We again used bivariate plots (Figure S6) to compare *BIOCLIM* MAP and MPET with average MAP and MPET values derived from Station + CRU for the 59 sites with both. *BIOCLIM* MAP and MPET were highly correlated ($R^2 > 0.95$) and unbiased (slope = 1.04) representations of Station + CRU MAP and were a small underestimation of Station + CRU mPET (slope = 0.91). Combining Station, CRU, and *BIOCLIM* precipitation and PET data did not introduce any substantive bias that might influence fertilization effects on biomass-MAP relationships.

Table S7. Summary of precipitation/PET data source correspondence with experimental aboveground biomass/diversity data years.

Obs	Site	Data Source	Number of aboveground biomass years	Number of Station + CRU data years
1	cdcr.us	Station	14	14
2	sgs.us	Station	14	14
3	cowi.ca	Station	14	13
4	hopl.us	Station	13	13
5	mtca.au	Station	13	13
6	sier.us	Station	13	13
7	spin.us	Station	14	13
8	cbgb.us	Station	12	12
9	elliot.us	Station	12	12
10	mcla.us	Station	13	12
11	bogong.au	Station	14	11
12	burrawan.au	Station	11	11
13	cdpt.us	Station	14	11
14	konz.us	Station	11	11
15	sevi.us	Station	15	11
16	temple.us	Station	11	11
17	kiny.au	Station	10	10
18	sedg.us	Station	10	10
19	bnch.us	CRU	14	9
20	koffler.ca	Station	11	9
21	lancaster.uk	Station	9	9
22	look.us	CRU	14	9
23	smith.us	Station	9	9
24	trel.us	Station	9	9
25	ukul.za	Station	11	9
26	valm.ch	Station	12	9
27	bldr.us	Station	8	8
28	jena.de	Station	9	8
29	ping.au	Station	8	8
30	saline.us	Station	8	8
31	doane.us	Station	7	7
32	frue.ch	Station	7	7
33	hall.us	Station	7	7
34	kilp.fi	Station	7	7
35	marc.ar	Station	11	7
36	saana.fi	Station	8	7
37	yarra.au	Station	7	7

**38**	arch.us	Station	7	6
**39**	badlau.de	Station	7	6
**40**	comp.pt	Station	9	6
**41**	ethamc.au	Station	6	6
**42**	ethass.au	Station	6	6
**43**	nilla.au	Station	6	6
**44**	pape.de	CRU	6	6
**45**	sage.us	Station	6	6
**46**	chilcas.ar	Station	9	5
**47**	hart.us	Station	5	5
**48**	hero.uk	Station	5	5
**49**	kbs.us	Station	6	5
**50**	rook.uk	Station	5	5
**51**	sava.us	Station	5	5
**52**	shps.us	Station	5	5
**53**	burren.ie	Station	5	4
**54**	cereep.fr	CRU	8	4
**55**	msla.us	Station	4	4
**56**	msla_2.us	Station	4	4
**57**	msla_3.us	Station	4	4
**58**	sereng.tz	CRU	4	4
**59**	unc.us	Station	4	4
**60**	bayr.de	BIOCLIM	5	3
**61**	gilb.za	BIOCLIM	4	3
**62**	pinj.au	BIOCLIM	4	3
**63**	ahth.is	BIOCLIM	4	0
**64**	amlr.is	BIOCLIM	4	0
**65**	azitwo.cn	BIOCLIM	4	0
**66**	bnbt.us	BIOCLIM	5	0
**67**	kibber.in	BIOCLIM	5	0
**68**	lagoas.br	BIOCLIM	8	0
**69**	lake.us	BIOCLIM	4	0
**70**	msum.us	BIOCLIM	5	0
**71**	potrok.ar	BIOCLIM	6	0

Figure S5. Comparison of station measured with CRU dataset values for annual precipitation (left) and potential evapotranspiration (PET, right). Solid lines and statistics are for linear regression fits. Dotted lines indicate 95% confidence intervals. Data points in magenta are for the sites where CRU either overestimated or underestimated station values; in these cases we retained the source with the lower values.

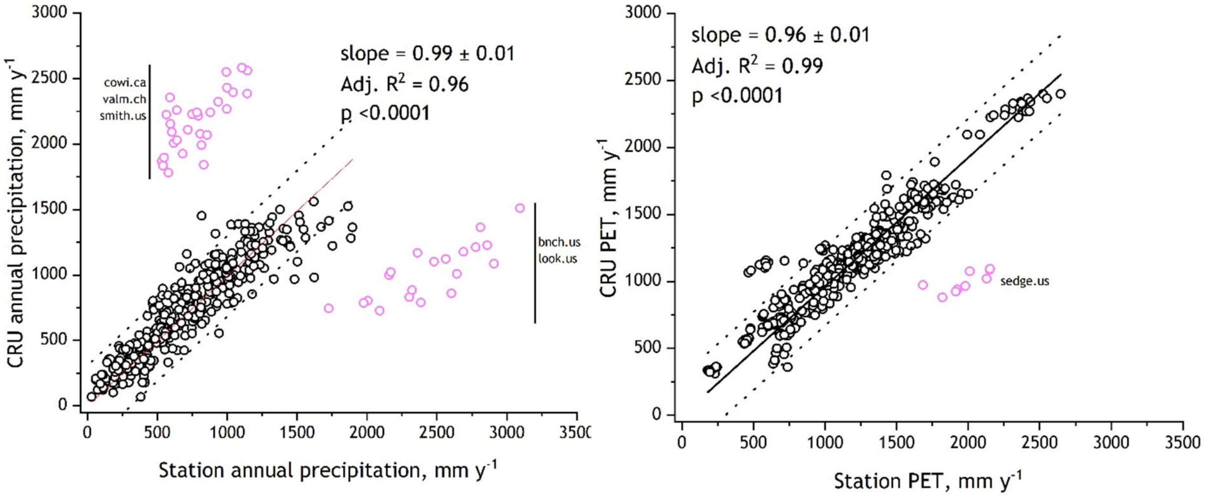

Figure S6. Comparison of mean annual precipitation (MAP, left) and mean annual potential evapotranspiration (MPET, right) when sourced from Station+CRU (horizontal axes) or *BIOCLIM* (vertical axes). Solid lines and statistics are for linear regression fits. Dotted lines indicate 95% confidence intervals.

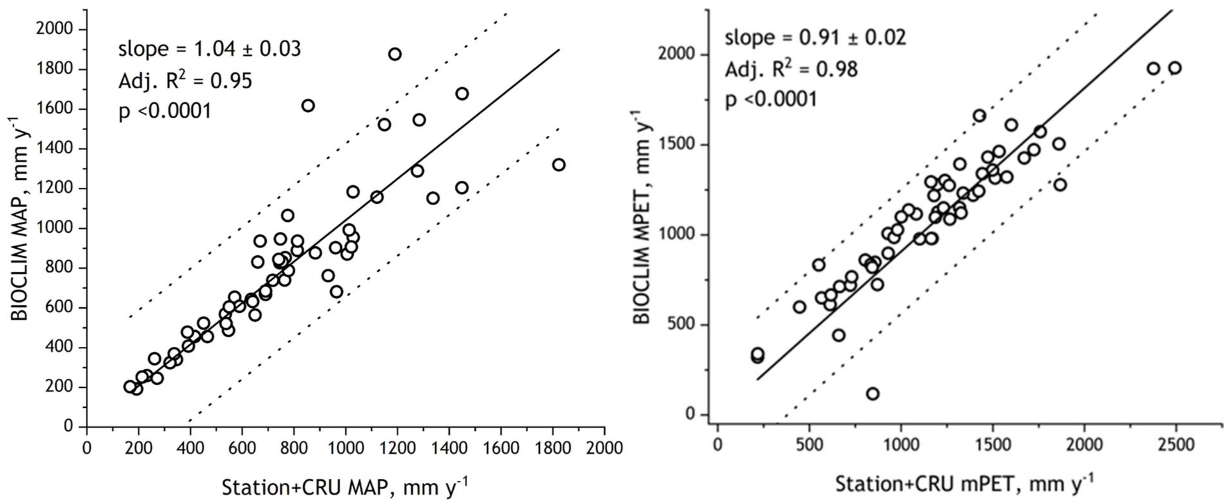

Screening Site Climate Variables. Our primary objective was to evaluate the role of site mean annual precipitation (MAP) in grassland responses to fertilization with single and multiple nutrients. We chose MAP after screening a set of 32 candidate site level mean climate variables related to mean precipitation supply and evaporative demand (Table S8). The climate variables tested included site-level MAP, MPET, MPET – MAP, and MPET*MAP^{-1}. We also screened climate variables included in the Nutrient Network dataset describing mean annual, quarterly, and monthly precipitation, temperature, and temperature or evaporative demand metrics relative to precipitation included in the Nutrient Network dataset, mostly from WORLDCLIM.

We screened the 32 candidate climate variables by evaluating model fit in a linear mixed model predicting aboveground biomass as a function of the factorial NPK fertilization treatments, climate variables, and climate variable x treatment interactions (Equation 1).

$$\text{aboveground biomass} = \mu + \text{nutrients} + \text{nutrients*climvar} + \text{error}. \qquad \text{(Equation 1)}$$

In Equation 1, *Nutrients* refers to all main effect and interaction terms for the fertilization treatments (e.g., N, P, NxP, Kμ, NxKμ, etc.), and *climvar* refers to a candidate climate variable. Random effects Site, block*site, and nutrients*site were also fit.

Equation 1 was fit in Proc Mixed in SAS STAT version 15.3 coded as:

```
Proc mixed method=reml covtest ic;
Class nutrients site block;
Model aboveground biomass= n|p|k|climvar/ddfm=kr;                (Model 1)
        Random site   block*site    n*p*k*site;
```

We fit Model 1 to aboveground biomass averaged at each site by block and fertilizer treatment across years to correspond in temporal scale to the site mean climate variables. The climate variables and aboveground biomass were centered and scaled (mean=0, standard deviation=1). The models were ranked by their AIC score (Table S8). MAP yielded the lowest AIC value (2175.3), which was lower than all other tested climate variables and lower than the model fit with no climate variable (AIC = 2190). Notably, many of the climate variables yielded worse model fit than the model with no climate variable. The climate variables other than MAP were not considered further.

Table S8. Akaike's Information Criterion (AIC) for 32 candidate climate variables describing mean site precipitation (MAP), temperature, potential evapotranspiration (PET), and temperature or PET relative to precipitation. Models are reported in order of increasing AIC within categories

Climate Variable	Definition	AIC
	**Precipitation Metrics**	
**MAP_mm**	Mean Annual Precipitation (Precipitation Dataset)	2175.3
**MAP_V2**	Mean Annual Precipitation (WorldClim)	2194.2
**MAP_WARM_Q_v2**	Precipitation of Warmest Quarter (WorldClim)	2197.0
**MAP_DRY_Q_v2**	Precipitation of Driest Quarter (WorldClim)	2199.2
**MAP_DRY_M_v2**	Precipitation of Driest Month (WorldClim)	2199.9
**MAP_WET_Q_v2**	Precipitation of Wettest Quarter (WorldClim)	2210.1
**MAP_WET_M_v2**	Precipitation of Wettest Month (WorldClim)	2212.1
**MAP_VAR_v2**	Precipitation Seasonality (Coefficient of Variation) (WorldClim)	2220.5
**MAP_COLD_Q_v2**	Precipitation of Coldest Quarter (WorldClim)	2220.9
	**Temperature Metrics**	
**ISO_v2**	Isothermality (Diurnal Range/Annual Range) (* 100) (WorldClim)	2207.6
**TEMP_VAR_v2**	Temperature Seasonality (standard deviation *100) (WorldClim)	2209.0
**TEMP_WARM_Q_v2**	Mean Temperature of Warmest Quarter (WorldClim)	2211.3
**ANN_TEMP_RANGE_v2**	Temperature Annual Range (WorldClim)	2214.1
**TEMP_WET_Q_v2**	Mean Temperature of Wettest Quarter (WorldClim)	2214.5
**MAX_TEMP_v2**	Max Temperature of Warmest Month (WorldClim)	2216.1
**MAT_RANGE_v2**	Mean Diurnal Range (Mean of monthly (max temp - min temp)) (WorldClim)	2216.4
**MAT_v2**	Annual Mean Temperature (BIOCLIM)	2216.5
**MIN_TEMP_v2**	Min Temperature of Coldest Month (WorldClim)	2216.5
**TEMP_Cold_Q_v2**	Mean Temperature of Coldest Quarter (WorldClim)	2217.0
**TEMP_DRY_Q_v2**	Mean Temperature of Driest Quarter (WorldClim)	2219.4
	**Evaporative demand indices relative to precipitation indices**	
**Maxv2_MAPmm**	MAX_v2 * MAP_mm-1	2192.2
**MPETmm_MAPmm**	MPET_mm * MAP_mm-1	2195.0
**defic_mm**	MPET_mm - MAP_mm	2199.9
**MATv2_MAPmm**	MAX_TEMP_v2 * MAP_mm-1	2203.9
**TtoP_COLDQ**	TEMP_COLD_Q_V2 * MAP_COLD_Q_V2-1	2205.9
**RAIN_PET**	Rainfall - potential evapotranspiration	2207.2
**TtoP_WETQ**	TEMP_WET_Q_V2 * MAP_WET_Q_V2-1	2210.1
**MPET_mm**	Mean Annual Potential Evapotranspiration (Precipitation Dataset)	2219.4
**AI**	Aridity Index (MAP / Mean annual PET) (CGIAR)	2220.9
**PET**	Potential Evapo-transpiration (mm yr-1) (CGIAR)	2222.8
**TtoP_WARMQ**	TEMP_WARM_Q_V2 * MAP_WARM_Q_V2-1	2223.3
**TtoP_DRYQ**	TEMP_DRY_Q_V2 * MAP_DRY_Q_V2-1	2225.1

SI References

1. W. S. Harpole *et al.*, Nutrient co-limitation of primary producer communities. *Ecol. Lett.* **14**, 852-862 (2011).
2. D. Hooper, J. Coughlan, M. Mullen, Structural equation modelling: guidelines for determining model fit. *Electron. J. Bus. Res. Methods* **6**, 53-60 (2008).
3. P. Droogers, R. G. Allen, Estimating Reference Evapotranspiration Under Inaccurate Data Conditions. *Irrigation and Drainage Systems* **16**, 33-45 (2002).
4. S. Bharath *et al.*, Nutrient addition increases grassland sensitivity to droughts. *Ecology* **101**, e02981 (2020).
5. S. E. Fick, R. J. Hijmans, WorldClim 2: new 1-km spatial resolution climate surfaces for global land areas. *International Journal of Climatology* **37**, 4302-4315 (2017).
